# Infection-experienced HSPCs protect against infections by generating neutrophils with enhanced mitochondrial bactericidal activity

**DOI:** 10.1126/sciadv.adf9904

**Published:** 2023-09-06

**Authors:** Hannah Darroch, Pramuk Keerthisinghe, Yih Jian Sung, Leah Rolland, Anneke Prankerd-Gough, Philip S. Crosier, Jonathan W. Astin, Christopher J. Hall

**Affiliations:** Department of Molecular Medicine and Pathology, Faculty of Medical and Health Sciences, University of Auckland, Auckland, New Zealand.

## Abstract

Hematopoietic stem and progenitor cells (HSPCs) respond to infection by proliferating and generating in-demand neutrophils through a process called emergency granulopoiesis (EG). Recently, infection-induced changes in HSPCs have also been shown to underpin the longevity of trained immunity, where they generate innate immune cells with enhanced responses to subsequent microbial threats. Using larval zebrafish to live image neutrophils and HSPCs, we show that infection-experienced HSPCs generate neutrophils with enhanced bactericidal functions. Transcriptomic analysis of EG neutrophils uncovered a previously unknown function for mitochondrial reactive oxygen species in elevating neutrophil bactericidal activity. We also reveal that driving expression of zebrafish C/EBPβ within infection-naïve HSPCs is sufficient to generate neutrophils with similarly enhanced bactericidal capacity. Our work suggests that this demand-adapted source of neutrophils contributes to trained immunity by providing enhanced protection toward subsequent infections. Manipulating demand-driven granulopoiesis may provide a therapeutic strategy to boost neutrophil function and treat infectious disease.

## Teaser

Demand-adapted hematopoiesis equips the host with neutrophils that kill bacteria faster through mitochondrial ROS production.

## INTRODUCTION

We now know that innate immunity has the ability to “remember” previous microbial encounters and generate stronger responses to subsequent infections with the same or unrelated pathogen (*1*–*3*). This memory function, termed trained immunity, is underpinned by epigenetic and metabolic changes within innate immune cells that support elevated antimicrobial responses. The longevity of trained immunity is the result of similar infection-induced changes within hematopoietic stem and progenitor cells (HSPCs) that provides a long-term supply of trained innate immune cells (*4*–*7*). The adaptive ability of HSPCs to sense bacterial threats and generate in-demand innate immune cells (termed demand-adapted hematopoiesis) is now recognized as an integral component of trained immunity. Further connecting demand-adapted hematopoiesis and trained immunity is the transcription factor CCAAT/enhancer binding protein beta (C/EBPβ), a central regulator of emergency granulopoiesis (EG; the production of in-demand neutrophils following infection) that has recently been shown to also control the epigenetic reprogramming of HSPCs during trained immunity (*8*–*11*).

To date, studies describing trained immunity have focused almost exclusively on monocytes/macrophages. Being the most abundant circulating leukocyte, neutrophils provide the essential first line of defense against microbial challenges. Fundamental neutrophil effector functions include phagocytosis and the subsequent destruction of intracellular bacteria through reactive oxygen species (ROS) production (*12*). Only recently have neutrophils been shown to contribute to trained immunity. Neutrophils from humans vaccinated with BCG (a driver of trained immunity) have been shown to respond more efficiently to microbial challenge (*13*). Furthermore, treating mice with β-glucan (another agonist of trained immunity) skews hematopoiesis toward the production of neutrophils with enhanced antitumor activity (*14*). Although these studies have revealed neutrophils as mediators of trained immunity, mechanisms that instruct their trained phenotypes remain poorly defined. Furthermore, whether EG contributes to trained immunity through generating neutrophils with heightened antibacterial responses is unknown.

Equipped with a highly conserved innate immune system and supported by neutrophil- and macrophage-marking transgenic reporter lines, transparent larval zebrafish provide a powerful system to directly observe the host response to microbial infection, at the single-cell level (*15*, *16*). Larval zebrafish can also model EG, where large numbers of neutrophils are produced within larval hematopoietic sites, the aorta-gonad-mesonephros (AGM) and caudal hematopoietic tissue (CHT), following infection (*17*–*21*). In addition, their exclusively innate immune system when at larval stages has been exploited to model trained immunity (*19*, *22*). We, and others, have shown that larval zebrafish HSPCs demonstrate a conserved response toward a sublethal bacterial challenge by expressing the zebrafish ortholog of *C/EBP*β (*cebpb*), expanding in number and enhancing their contribution toward the neutrophil lineage (*17*, *19*). Recently, we showed that this EG infection model can drive an overlapping trained immune response (*22*). A feature of our EG model was a cohort of infected larvae that became almost completely neutropenic before de novo neutrophil production (*17*). We speculated that this would facilitate the study of EG-generated neutrophils in isolation from those generated under steady-state (SS) conditions.

Here, we provide evidence that infection-experienced HSPCs drive an EG response that populates the host with neutrophils equipped with enhanced bactericidal functions. Our work suggests that this demand-adapted source of neutrophils likely contributes to trained immunity through providing enhanced heterologous protection toward subsequent bacterial challenges.

## RESULTS

### Larvae populated with EG neutrophils demonstrate enhanced survival to subsequent infections

**Fig. 1.**
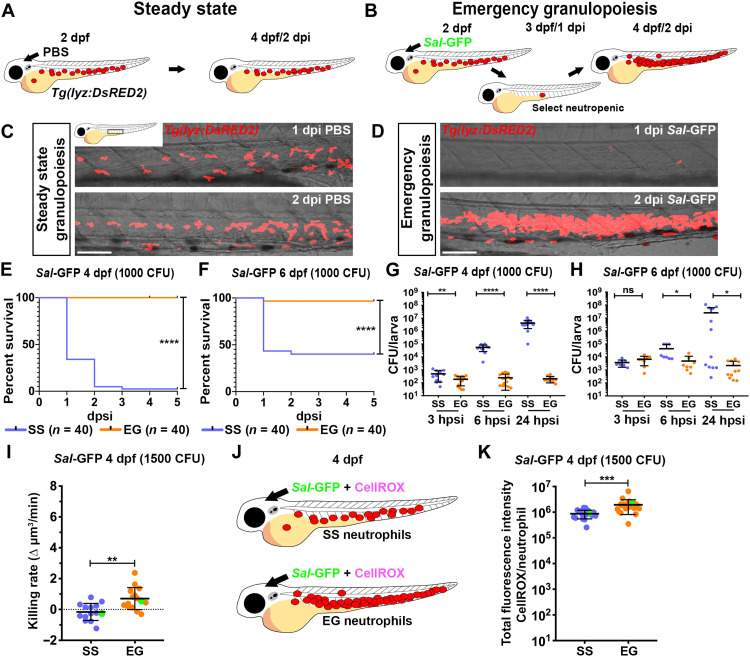
EG larvae have elevated survival to subsequent bacterial challenge and have neutrophils with enhanced bactericidal activity. (**A** and **B**) Schematic illustrating strategy to generate SS (A) and EG (B) larvae. (**C** and **D**) Live imaging of the AGM/CHT regions within *Tg(lyz:DsRED2)* larvae at 1 and 2 dpi with PBS (C) or *Sal*-GFP (D). (**E** and **F**) Kaplan-Meier graphs showing survival of SS and EG larvae over 5 dpsi with *Sal*-GFP at 4 dpf (E) and 6 dpf (F). (**G** and **H**) Bacterial burdens within individual SS or EG larvae at 3, 6, and 24 hpsi with *Sal*-GFP at 4 dpf (G) and 6 dpf (H). (**I**) Bacterial killing rates of SS and EG neutrophils following *Sal*-GFP infection. Green data points highlight killing rates of neutrophils as shown in fig. S1D. (**J**) Schematic illustrating injection of SS and EG larvae with *Sal*-GFP and CellROX. (**K**) Quantification of ROS production within *Sal*-GFP-laden SS and EG neutrophils, as detected by CellROX fluorescence. Green data points highlight ROS production of neutrophils as shown in fig. S1G. Error bars, mean ± SD; ns, not significant, *P < 0.05, **P < 0.01, ***P < 0.001, ****P < 0.0001; Gehan-Breslow-Wilcoxon test (E and F) and unpaired Student’s *t* test (G, H, I, and K). Scale bars, 50 μm.

To generate larvae populated with EG neutrophils, 2 day postfertilization (dpf) neutrophil-marking *Tg(lyz:DsRED2)* larvae (*23*) were injected with a sublethal dose [600 colony-forming units (CFU)] of green fluorescent protein (GFP)–expressing *Salmonella enterica* (hereafter referred to as *Sal-*GFP) into the hindbrain ventricle, as previously described (*17*). Infected larvae were then manually inspected for neutropenia at 1 day postinjection (dpi) by fluorescence microscopy (Fig. 1B). Here, neutropenic larvae are defined as those that have no more than 15 to 20 remaining neutrophils, which equates to 8.4 ± 2.6% (mean ± SD) of SS neutrophil numbers when quantified by flow cytometry (fig. S1A). Larvae with high neutrophil abundance, or that retained residual *Sal*-GFP (as assessed by fluorescence microscopy), were discarded. Larvae that underwent EG were selected at 2 dpi (4 dpf) by identifying those with de novo neutrophil production within the AGM and CHT (Fig. 1D). On average, 27.9 ± 1.5% (mean ± SD, *n* = ~100 larvae in biological triplicate) of infected larvae became neutropenic, and of those larvae 74.5 ± 3.9% demonstrated strong EG. To generate larvae populated with SS neutrophils, 2 dpf *Tg(lyz:DsRED2)* larvae were injected with phosphate-buffered saline (PBS) (Fig. 1, A and C). Similar to our previous study (*17*), larvae populated with EG neutrophils had approximately 2.5-fold more neutrophils than PBS-injected controls, with numbers returning to SS levels by 7 dpi (fig. S1A). Throughout the remainder of this study, larvae populated with SS and EG neutrophils are referred to as SS and EG larvae, respectively.

To initially investigate differences between SS and EG neutrophils, we infected SS and EG larvae and monitored their survival. SS or EG larvae were infected with a lethal dose (1000 CFU) of *Sal*-GFP into the hindbrain ventricle at 4 dpf and monitored for 5 days postsecondary injection (dpsi). This analysis revealed that EG larvae had a significant survival advantage that was associated with enhanced bacterial clearance (Fig. 1, E and G). Furthermore, the enhanced antibacterial response of EG larvae was maintained when extending the interval between primary and secondary infections to 4 days (Fig. 1, F and H). Notably, despite EG larvae having more neutrophils than SS larvae, similar numbers were recruited to the infection site (fig. S1, A to C).

### EG neutrophils have enhanced bactericidal capacity and generate more ROS when compared to SS neutrophils

We next investigated whether EG neutrophils displayed functional differences that could, at least in part, contribute to the elevated survival demonstrated by EG larvae. We used a method we previously developed to measure neutrophil bacterial killing capacity in larval zebrafish through live time-lapse confocal imaging of *Sal*-GFP-laden neutrophils and measuring the change in intracellular bacterial volume (as detected by *Sal*-GFP fluorescence) over time (“killing rate” = Δμm^3^/time) (*24*). SS and EG larvae were infected with 1500 CFU *Sal*-GFP at 4 dpf into the hindbrain ventricle and imaged from 2 to 4 hours postsecondary injection (hpsi). This analysis revealed that EG neutrophils demonstrated significantly higher killing rates when compared to SS neutrophils (Fig. 1I and fig. S1D). To assess whether this enhanced bactericidal activity of EG neutrophils was heterologous in nature, we next measured the killing rates of SS and EG neutrophils when challenged with another pathogen, GFP-expressing Gram-positive *Streptococcus iniae*. Similar to their response to Gram-negative *Sal*-GFP, EG neutrophils killed intracellular *S. iniae* faster than SS neutrophils (fig. S1, E and F). We then investigated whether EG neutrophils produce more ROS when compared to SS neutrophils by coinjecting the ROS-sensitive fluorescent probe CellROX with *Sal*-GFP at 4 dpf (Fig. 1J), as previously described (*24*). This analysis revealed that EG neutrophils produced significantly higher levels of ROS, as detected by CellROX fluorescence intensity (Fig. 1K and fig. S1G). When live imaging neutrophil bactericidal activity, we could not discount the possibility that some cells we classified as EG neutrophils may be the few remaining neutrophils we sometimes observed when scoring for neutropenia at 1 dpi (that could have been generated under SS conditions). To help enrich for EG neutrophils, we used the *Tg(mpx:Dendra2)* neutrophil reporter line (*25*) to specifically photoconvert neutrophils within the CHT following EG and measure their killing rates following infection. A 405-nm laser was used to photoconvert Dendra2-expressing neutrophils from green to red within the CHT of SS and EG larvae before infection (fig. S2, A and B). Photoconverted EG neutrophils had significantly elevated bacterial killing rates when compared to photoconverted SS neutrophils, supporting our earlier results (fig. S2, C and D). Collectively, these data reveal that neutrophils within infected EG larvae produce more ROS than those generated under SS conditions and can kill intracellular bacteria faster.

### EG neutrophils contribute to enhanced survival toward subsequent infection

Our data suggested that the survival advantage demonstrated by EG larvae was, at least in part, due to the elevated bactericidal activity of EG neutrophils. To assess the contribution of EG neutrophils to larval survival, we next examined larval survival in the absence of EG neutrophils.

**Fig. 2.**
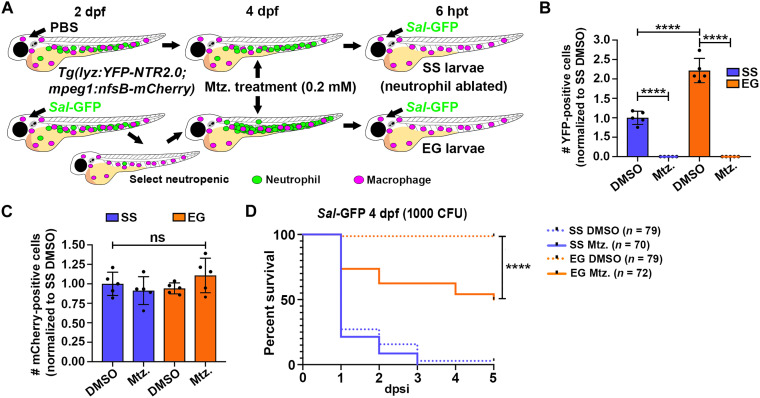
Ablating EG neutrophils reduces survival to subsequent infection. (**A**) Schematic illustrating strategy to ablate SS and EG neutrophils. *Tg(lyz:YFP-NTR2.0;mpeg1:nfsB-mCherry)* SS larvae and those demonstrating EG were treated with dimethyl sulfoxide (DMSO; control) or 0.2 mM mtz. At 6 hours posttreatment (hpt), neutrophil-ablated larvae were infected with *Sal*-GFP and treated continuously with 0.1 mM mtz. (**B**) Flow quantification of YFP-expressing neutrophils from whole SS and EG *Tg(lyz:YFP-NTR2.0;mpeg1:nfsB-mCherry)* larvae, 6 hours following mtz treatment, compared to DMSO-treated controls (*n* = 20 larvae per sample, five experimental replicates). (**C**) Flow quantification of mCherry-expressing macrophages from whole SS and EG *Tg(lyz:YFP-NTR2.0;mpeg1:nfsB-mCherry)* larvae, 6 hours following mtz treatment, compared to DMSO-treated controls (*n* = 20 larvae per sample, five experimental replicates). (**D**) Kaplan-Meier graph showing survival of DMSO- and mtz-treated SS and EG *Tg(lyz:YFP-NTR2.0;mpeg1:nfsB-mCherry)* larvae over 5 dpsi with *Sal*-GFP at 4 dpf. Error bars, mean ± SD; ****P < 0.0001; one-way analysis of variance (ANOVA) with Tukey’s multiple comparisons test (B and C) and Gehan-Breslow-Wilcoxon test (D).

To ablate neutrophils, we took advantage of a transgenic line we recently developed [*Tg(lyz:YFP-NTR2.0)*] that uses a rationally engineered variant of nitroreductase (called NTR 2.0) to effect rapid and complete neutrophil ablation (*26*, *27*). Using metronidazole (mtz) concentrations 100-fold less than those previously used for first-generation NTR ablation lines, *YFP-NTR2.0*-expressing neutrophils within *Tg(lyz:YFP-NTR2.0)* larvae are ablated within hours (*26*). First, we developed an mtz treatment regimen to ablate SS and EG neutrophils before infection. Treating SS and EG *Tg(lyz:YFP-NTR2.0)* larvae for 6 hours with 0.2 mM mtz was sufficient to effect complete neutrophil ablation, as assessed by flow cytometry (Fig. 2, A and B). To investigate the specificity of ablation, we used the macrophage-marking *Tg(mpeg1:nfsB-mCherry)* line that expresses an mCherry-tagged first-generation NTR enzyme (encoded by the *Escherichia coli* gene *nfsB*) that requires much higher mtz doses for ablation (*28*, *29*). As expected, macrophage numbers remained unchanged in *Tg(lyz:YFP-NTR2.0;mpeg1:nfsB-mCherry)* SS and EG larvae following the same 0.2 mM mtz dose (Fig. 2C). This analysis also supported our previous work (*17*) by showing unchanged numbers of macrophages in EG larvae, supporting a neutrophil-specific demand-driven hematopoietic response in our infection model (Fig. 2C). Next, neutrophil-ablated *Tg(lyz:YFP-NTR2.0;mpeg1:nfsB-mCherry)* SS and EG larvae were infected and their survival followed in the presence of 0.1 mM mtz, a dose that maintains neutrophil ablation with no obvious toxicity (fig. S3A). Consistent with the enhanced bactericidal activity of EG neutrophils, larvae were more sensitive to infection in their absence (Fig. 2D). To exclude any possibility that our prolonged mtz treatment regimen was having an impact on the *nfsB-mCherry*–expressing macrophages used in our study (that may contribute toward the reduced survival observed in our mtz-treated EG larvae), we also assessed macrophage numbers within *Tg(lyz:YFP-NTR2.0;mpeg1:nfsB-mCherry)* larvae following the 5-day mtz treatment regimen and the survival of similarly treated *Tg(mpeg1:nfsB-mCherry)* larvae infected with *Sal*-GFP at 4 dpf. These analyses revealed that macrophage numbers remained unaltered at the completion of the treatment regimen and that there was no impact on macrophage bactericidal activity (fig. S3, B and C). These results confirm that EG neutrophils contribute toward the enhanced survival phenotype demonstrated by EG larvae following infection.

### EG neutrophils maintain elevated killing rates in macrophage-depleted larvae and when transplanted into infection-naïve recipients

**Fig. 3.**
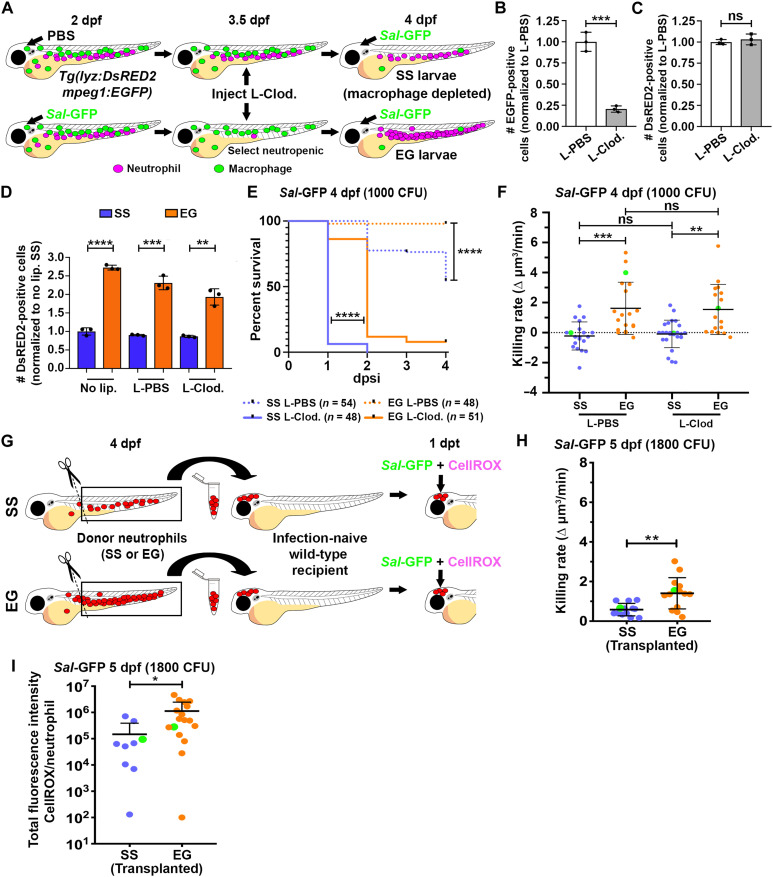
EG neutrophils maintain elevated killing rates in macrophage-depleted larvae and when transplanted into infection-naïve recipients. (**A**) Schematic illustrating strategy to deplete macrophages within SS and EG larvae. (**B** and **C**) Flow quantification of EGFP-expressing macrophages (B) and DsRED2-expressing neutrophils (C) from whole 4 dpf *Tg(lyz:DsRED2;mpeg1:EGFP)* larvae, following liposomal-clodronate (L-Clod) injection, compared to liposomal-PBS (L-PBS) injection controls (*n* = 20 larvae per sample, three experimental replicates). (**D**) Flow quantification of DsRED2-expressing neutrophils from whole 4 dpf SS and EG *Tg(lyz:DsRED2;mpeg1:EGFP)* larvae, 1 day following L-Clod. injection, compared to L-PBS injection and no liposome (no lip.) controls (*n* = 20 larvae per sample, three experimental replicates). (**E**) Kaplan-Meier graph showing survival of SS L-PBS, SS L-Clod., EG L-PBS, and EG L-Clod. larvae over 4 dpsi with *Sal*-GFP at 4 dpf. (**F**) Bacterial killing rates of SS and EG neutrophils within L-Clod.– and L-PBS–injected *Tg(lyz:DsRED2;mpeg1:EGFP)* larvae following *Sal*-GFP infection. Green data points highlight killing rates of neutrophils as shown in fig. S4C. (**G**) Schematic illustrating transplantation of FACS-isolated SS and EG neutrophils into infection-naïve recipient larvae. (**H**) Bacterial killing rates of transplanted SS and EG neutrophils following *Sal*-GFP infection. Green data points highlight killing rates of neutrophils as shown in fig. S4D. (**I**) Quantification of ROS production within *Sal*-GFP-laden transplanted SS and EG neutrophils, as detected by CellROX fluorescence. Green data points highlight ROS production of neutrophils as shown in fig. S4E. Error bars, mean ± SD; *P < 0.05, **P < 0.01, ***P < 0.001, ****P < 0.0001; unpaired Student’s *t* test (B, C, D, H, and I), Gehan-Breslow-Wilcoxon test (E), and one-way ANOVA with Tukey’s multiple comparisons test (F).

Given that infected EG larvae still maintained a survival advantage in the absence of neutrophils (Fig. 2D), our neutrophil ablation studies suggested that other factors contribute to the enhanced survival observed for EG larvae. Given that macrophages are well known to undergo training and can directly influence neutrophil function (*30*, *31*), we next investigated the survival of EG larvae in the absence of macrophages, and whether the elevated bactericidal activity of EG neutrophils was dependent on macrophages. To remove macrophages, we injected liposomal-clodronate (*29*) into SS and EG larvae (at the neutropenic stage) at 3.5 dpf, before *Sal*-GFP infection at 4 dpf (Fig. 3A). This treatment resulted in ~80% macrophage depletion at 4 dpf (Fig. 3B) while leaving neutrophil numbers (Fig. 3C) and the EG response (Fig. 3D) unaffected. As expected, macrophage-depleted larvae were more sensitive to *Sal*-GFP infection (Fig. 3E). However, macrophage-depleted EG larvae still maintained a survival advantage, when compared to macrophage-depleted SS larvae (Fig. 3E), a phenotype supported by significantly enhanced bacterial clearance at 9 hpsi (fig. S4, A and B). Examining neutrophil killing rates in macrophage-depleted EG larvae revealed that macrophages were dispensable for the enhanced bactericidal activity of EG neutrophils (Fig. 3F and fig. S4C).

When quantifying the bacterial killing capacity and ROS production of EG neutrophils, we were unsure whether these enhanced effector functions were a cell-intrinsic feature of these newly generated neutrophils or the result of being within a previously infected host where unknown infection-responsive mechanisms may contribute. To address this, we exploited a neutrophil transplantation protocol we previously developed (*32*) to examine whether the observed elevated killing rates and ROS production of EG neutrophils were maintained following transplantation into an infection-naïve host. SS and EG neutrophils were isolated from the dissected trunks (containing the AGM and CHT) of SS and EG larvae, respectively, and injected into the hindbrain ventricle of age-matched, infection-naïve recipients (Fig. 3G). At 1 day posttransplant (dpt), recipients were infected with 1800 CFU *Sal*-GFP, and the bacterial killing rates and ROS production within individual transplanted SS or EG neutrophils were quantified. Transplanted EG neutrophils maintained significantly elevated bacterial killing rates and ROS production when compared to transplanted SS neutrophils (Fig. 3, H and I, and fig. S4, D and E). These results strongly suggest that the enhanced bactericidal activity of EG neutrophils is not dependent on macrophages and is a cell-intrinsic feature of these newly generated neutrophils.

### EG neutrophils have elevated expression of mitochondria-associated genes following infection and use mtROS for their enhanced bactericidal activity

**Fig. 4.**
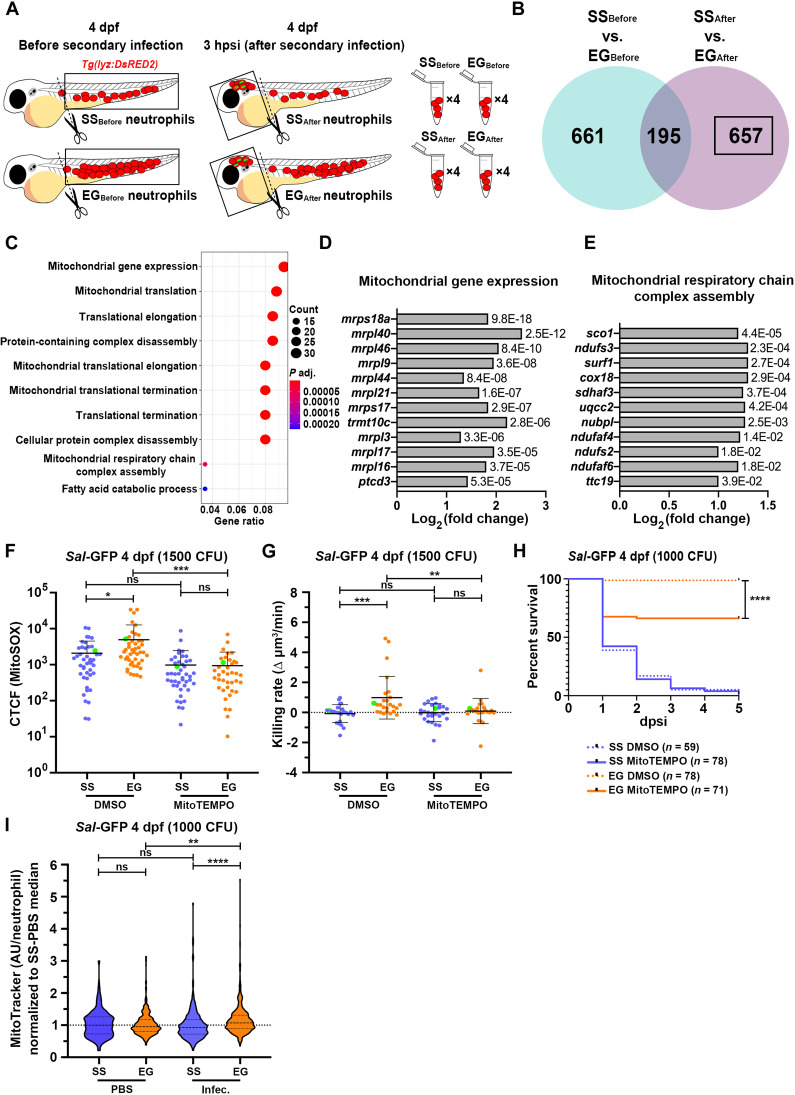
EG neutrophils enhance expression of mitochondria-associated genes following infection, use mtROS for their enhanced bactericidal activity and have greater mitochondrial mass. (**A**) Schematic illustrating sample collection for RNA-seq of SS and EG neutrophils before (cells harvested from dissected trunks) and after (cells harvested from dissected heads) infection. (**B**) Venn diagram of DEGs up-regulated in EG neutrophils that were unique to the “SS_Before_ versus EG_Before_” and “SS_After_ versus EG_After_” pairwise comparisons and those common to both comparisons. Black box highlights DEGs of interest. (**C**) GO:BP analysis of DEGs of interest. (**D**) Log_2_ fold change of genes associated with the “mitochondrial gene expression” GO term, with adjusted *P* values. (**E**) Log_2_ fold change of genes associated with the “mitochondrial respiratory chain complex assembly” GO term, with adjusted *P* values. (**F**) Quantification of mtROS within *Sal*-GFP-laden SS and EG neutrophils in the presence of MitoTEMPO or DMSO (control), as detected by MitoSOX fluorescence. Green data points highlight mtROS within neutrophils as shown in fig. S5A. (**G**) Bacterial killing rates of SS and EG neutrophils in the presence of MitoTEMPO or DMSO (control) following *Sal*-GFP infection. Green data points highlight killing rates of neutrophils as shown in fig. S5B. (**H**) Kaplan-Meier graph showing survival of DMSO- and MitoTEMPO-treated SS and EG larvae over 5 dpsi with *Sal*-GFP at 4 dpf. (**I**) Flow quantification of mitochondrial mass [as measured with MitoTracker relative fluorescence intensity in arbitrary units (AU)/neutrophil] within individual neutrophils from SS and EG larvae, following *Sal*-GFP infection at 4 dpf, compared to PBS-injected controls. Error bars, mean ± SD; *P < 0.05, **P < 0.01, ***P < 0.001, ****P < 0.0001; one-way ANOVA with Tukey’s multiple comparisons test (F, G, and I) and Gehan-Breslow-Wilcoxon test (H).

Given that our neutrophil transplantation experiments strongly suggested that the enhanced capacity of EG neutrophils to kill intracellular bacteria was cell intrinsic, we predicted that transcriptional changes may underpin this augmented activity. Bulk RNA sequencing (RNA-seq) was performed on SS and EG neutrophils before (SS_Before_ and EG_Before_) and after (SS_After_ and EG_After_) infection (Fig. 4A). Differentially expressed genes (DEGs) were then identified from pairwise comparisons (Fig. 4B). We focused on DEGs that were up-regulated in EG_After_ neutrophils when compared to SS_After_ neutrophils as these groups were a direct representation of the neutrophils we functionally characterized in our prior experiments. More specifically, we focused on the 657 genes that were exclusively up-regulated after infection in EG_After_ neutrophils when compared to SS_After_ neutrophils (Fig. 4B). Gene ontology enrichment analysis focusing on biological processes (GO:BP) performed on this gene set (DEGs of interest) revealed that mitochondrial gene expression and mitochondrial respiratory complex assembly were significantly enriched GO terms (Fig. 4, C to E). Genes that contributed to the mitochondrial respiratory complex assembly pathway included subunits and assembly factors of electron transport chain (ETC) complex 1 (*ndufs3*, *ndufs2*, *ndufaf6*, *nubpl*, and *ndufaf4*) and complex III (*uqcc2* and *ttc19*) (Fig. 4E). Notably, ETC complex I and III are specifically involved in mitochondrial ROS (mtROS) generation (*33*). Furthermore, mitochondria have been shown to provide a source of bactericidal ROS to kill intracellular bacteria within phagocytic cells (*34*–*38*).

**Fig. 5.**
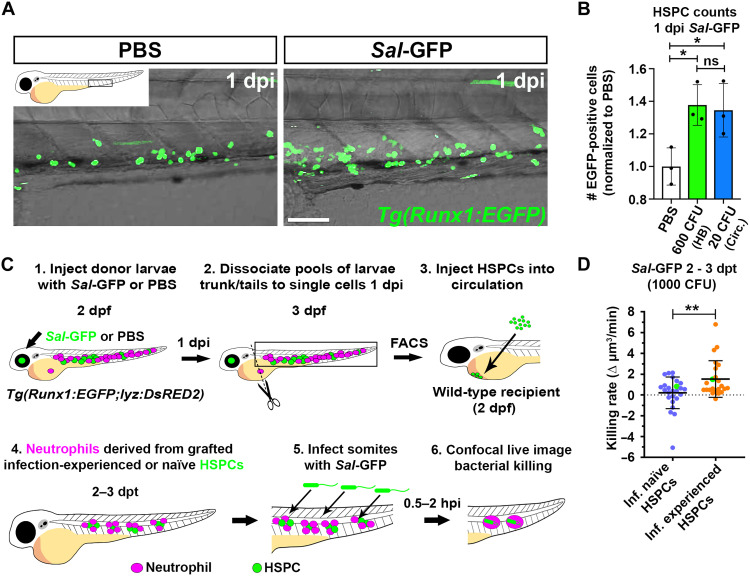
Infection-experienced HSPCs generate neutrophils with elevated bactericidal activity. (**A**) Live confocal imaging of HSPCs in the CHT of 3 dpf *Tg(Runx1:EGFP)* larvae, 1 day following injection with PBS or *Sal*-GFP. (**B**) Flow quantification of HSPCs from dissected trunks of *Tg(Runx1:EGFP)* larvae 1 day following injection with PBS or *Sal-*GFP into the hindbrain ventricle (HB) or circulation (Circ.), *n* = 20 larvae per sample, three experimental replicates. (**C**) Schematic illustrating transplantation strategy to live image neutrophils derived from infection-naïve and infection-experienced HSPCs. (**D**) Bacterial killing rates of neutrophils derived from infection-naïve and infection-experienced HSPCs. Green data points highlight killing rates of neutrophils as shown in fig. S6H. Error bars, mean ± SD; *P < 0.05, **P < 0.01; one-way ANOVA with Tukey’s multiple comparisons test (B) and unpaired Student’s *t* test (D). Scale bar, 50 μm.

To investigate whether mtROS was contributing to the increased bacterial killing capacity of EG neutrophils, SS and EG *Tg(lyz:EGFP)* larvae were coinjected with 1500 CFU *Sal*-GFP and the mitochondria-specific fluorescent superoxide indicator MitoSOX Red at 4 dpf. Quantification of mtROS within *Sal*-GFP-laden SS and EG neutrophils revealed that EG neutrophils produced significantly greater amounts of mtROS when compared to SS neutrophils (Fig. 4F and fig. S5A). This increase in mtROS was abolished in the presence of the mitochondria-targeted antioxidant MitoTEMPO (Fig. 4F and fig. S5A). Supporting the bactericidal activity of this mtROS, the increased bacterial killing capacity of EG neutrophils was not observed following MitoTEMPO treatment (Fig. 4G and fig. S5B). Furthermore, MitoTEMPO treatment significantly blunted the survival advantage observed in EG larvae following infection (Fig. 5H). Closer examination of the 657 DEGs of interest revealed that *tfam*, a mitochondrial transcription factor that controls mitochondrial transcriptional machinery and regulates mitochondrial biogenesis (*39*, *40*), was exclusively up-regulated in EG neutrophils following infection (fig. S5C). In addition, the zebrafish ortholog of *TIMM23* (*timm23a*), another mitochondrial gene whose expression has been shown to correlate with mitochondrial content and mtROS production (*41*), was similarly up-regulated (fig. S5C). These results prompted us to examine the mitochondrial content of SS and EG neutrophils following infection. MitoTracker staining of SS and EG neutrophils, both before (PBS-injected) and after infection, revealed that EG neutrophils had elevated mitochondrial mass following infection (Fig. 4I). Together, these data suggest that EG neutrophils turn on an infection-responsive transcriptional program to elevate mtROS production that contributes toward their increased bactericidal activity.

### Infection-experienced HSPCs generate neutrophils with elevated bactericidal activity

Given demand-adapted hematopoiesis is known to be instructed at the level of HSPCs (*42*–*46*), we speculated that the enhanced bactericidal activity of EG neutrophils was the result of infection-induced changes within HSPCs. We developed an HSPC transplantation protocol to live image neutrophils derived from transplanted infection-experienced or infection-naïve HSPCs. To label HSPCs, we used the mouse *Runx1* +24 enhancer to recreate the *Tg(Mmu.Runx1:EGFP)* reporter line previously used to live image and quantify HSPCs (*47*, *48*), herein referred to as *Tg(Runx1:EGFP)*. Notably, the *Tg(Runx1:EGFP)* line also has the lens-marking *cry:GFP* transgenesis marker. To validate that our *Tg(Runx1:EGFP)* line marked HSPCs, live confocal imaging revealed that GFP-positive cells resided within the AGM and CHT, were small and spherical, and demonstrated “endothelial cuddling,” a unique interaction between HSPCs and endothelial cells that was first characterized within larval zebrafish (Fig. 5A and fig. S6A) (*49*). In addition, examination of fluorescence-activated cell sorting (FACS)–isolated GFP-positive cells from the dissected trunks of *Tg(Runx1:EGFP)* larvae showed that they resembled HSPCs with large nuclei and sparse ungranulated cytoplasm (*50*) and that they expressed the classical HSPC markers *runx1* and *cmyb* (fig. S6, B and C) (*51*, *52*). Consistent with our earlier work (*17*), HSPC abundance within *Tg(Runx1:EGFP)* larvae was significantly increased at 1 dpi following injection of *Sal*-GFP into both the hindbrain ventricle and the circulation (~38 and 35% increases for hindbrain and circulation, respectively) (Fig. 5, A and B).

To observe neutrophils generated from infection-experienced or infection-naïve HSPCs, HSPCs were FACS-isolated from 3 dpf *Tg(Runx1:EGFP;lyz:DsRED2)* larvae 1 day following injection with 600 CFU *Sal*-GFP (infection-experienced) or PBS (infection-naïve) (Fig. 5C). HSPCs were then transplanted into the circulation of 2 dpf infection-naïve wild-type recipients (Fig. 5C). To ensure that no residual *Sal*-GFP was injected alongside HSPCs, transplantation mixtures were plated (fig. S6D). Larvae were then screened for successful engraftment from 1 dpt by confocal microscopy. HSPCs homed to the CHT where they were observed to divide and contribute to the neutrophil lineage from 2 dpt (fig. S6, E to G). Successfully engrafted infection-naïve recipients that had DsRED2-positive neutrophils were then infected with 1000 CFU *Sal*-GFP, and neutrophil bacterial killing rates were quantified (Fig. 5C). Notably, given that infections were cleared too rapidly by host immunity when delivered into the hindbrain ventricle, infections were targeted to the somites, in close proximity to engrafted HSPCs and their DsRED2-positive neutrophil progeny. Neutrophils derived from infection-experienced HSPCs had significantly higher bacterial killing rates when compared to neutrophils derived from infection-naïve HSPCs (Fig. 5D and fig. S6H).

### Overexpressing *cebpb* within HSPCs is sufficient to drive EG and generate neutrophils with elevated bactericidal activity

Given the technically challenging nature of our HSPC transplantation technique, where only a small number of neutrophils were generated from transplanted cells, we were unable to explore whether the neutrophils derived from infection-experienced HSPCs used the same mitochondrial mechanism to boost their bactericidal activity. To circumvent this, given our previous work using the same infection model had shown that the zebrafish ortholog of C/EBPβ is expressed within Runx1-positive HSPCs 12 hours postinfection and drives the EG response (*17*), we next explored whether overexpressing *cebpb* in HSPCs was sufficient to enhance granulopoiesis and generate neutrophils with elevated bactericidal activity.

**Fig. 6.**
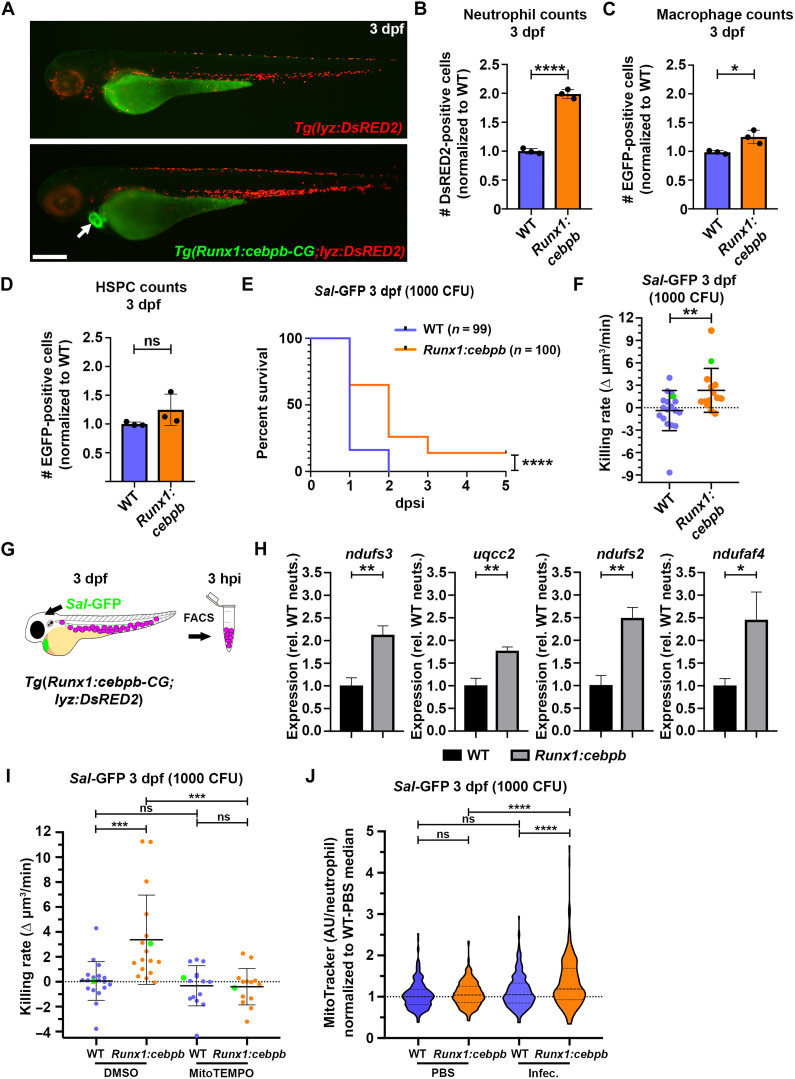
Larvae with *cebpb*-overexpressing HSPCs demonstrate enhanced granulopoiesis, survival to infection, and have neutrophils with enhanced bactericidal activity and mitochondrial mass. (**A**) Live imaging of *Tg(lyz:DsRED2)* and *Tg(Runx1:cebpb-CG2;lyzDsRED2)* larvae at 3 dpf. (**B**) Flow quantification of neutrophils from 3 dpf *Tg(lyz:DsRED2)*/WT and *Tg(Runx1:cebpb-CG2;lyzDsRED2)* larvae (*n* = 10 larvae per sample, three experimental replicates). (**C**) Flow quantification of macrophages from the dissected trunks of 3 dpf *Tg(mpeg1:EGFP)*/WT and *Tg(Runx1:cebpb-CG2;mpeg1:EGFP)* larvae (*n* = 10 larvae per sample, three experimental replicates). (**D**) Flow quantification of HSPCs from the dissected trunks of 3 dpf *Tg(Runx1:EGFP)*/WT and *Tg(Runx1:cebpb-CG2;Runx1:EGFP)* larvae, respectively (*n* = 25 to 50 larvae per sample, three experimental replicates). (**E**) Kaplan-Meier graph showing survival of *Tg(lyz:DsRED2)*/WT and *Tg(Runx1:cebpb-CG2;lyzDsRED2)* larvae over 5 dpsi with *Sal*-GFP at 3 dpf. (**F**) Bacterial killing rates of neutrophils within 3 dpf *Tg(lyz:DsRED2)*/WT and *Tg(Runx1:cebpb-CG2;lyzDsRED2)* larvae. Green data points highlight killing rates of neutrophils as shown in fig. S7C. (**G**) Schematic illustrating strategy to FACS-isolate neutrophils from infected *Tg(Runx1:cebpb-CG2;lyzDsRED2)* larvae. (**H**) Expression of *ndufs3*, *uqcc2*, *ndufs2*, and *ndufaf4* within neutrophils FACS-isolated from infected *Tg(lyzDsRED2)*/WT and *Tg(Runx1:cebpb-CG2;lyzDsRED2)* larvae [as shown in (G)], as detected by qPCR (in biological triplicate). (**I**) Bacterial killing rates of neutrophils within 3 dpf *Tg(lyz:DsRED2)*/WT and *Tg(Runx1:cebpb-CG2;lyzDsRED2)* larvae following MitoTEMPO and DMSO (control) treatment. Green data points highlight killing rates of neutrophils as shown in Fig. S7D. (**J**) Flow quantification of mitochondrial mass (as measured with MitoTracker relative fluorescence intensity in arbitrary units/neutrophil) within individual neutrophils from *Tg(lyz:DsRED2)*/WT and *Tg(Runx1:cebpb-CG2;lyzDsRED2)* larvae following *Sal*-GFP infection at 3 dpf, compared to PBS-injected controls. Error bars, mean ± SD; *P < 0.05, **P < 0.01, ***P < 0.001, ****P < 0.0001; unpaired Student’s *t* test (B, C, D, F, and H), Gehan-Breslow-Wilcoxon test (E), and one-way ANOVA with Tukey’s multiple comparisons test (I and J). Scale bar, 250 μm.

To investigate whether Cebpb was sufficient to drive EG within infection-naïve larvae, we generated a transgenic line overexpressing *cebpb* within HSPCs using the mouse *Runx1* +23 enhancer (*49*) that incorporated the heart-marking *cmlc2:GFP* (CG2) transgenesis marker (*53*), herein referred to as *Tg(Runx1:cebpb-CG2)* (fig. S7, A and B). Examining neutrophil abundance within *Tg(Runx1:cebpb-CG2;lyz:DsRed2)* larvae revealed a twofold increase at 3 dpi, when compared to *Tg(lyz:DsRed2*)/WT controls (Fig. 6, A and B). This was in contrast to macrophages and HSPCs, whose numbers remained largely unaltered, when quantified from the dissected trunks of *Tg(Runx1:cebpb-CG2;mpeg1:EGFP)* and *Tg(Runx1:cebpb-CG2;Runx1:EGFP)* larvae, respectively (Fig. 6, C and D). Similar to larvae populated with EG neutrophils, *Tg(Runx1:cebpb-CG2;lyz:DsRed2)* larvae had a survival advantage following infection, and their neutrophils could kill intracellular *Sal*-GFP faster than *Tg(lyz:DsRed2)*/WT neutrophils (Fig. 6, E and F, and fig. S7C).

To explore whether a similar mtROS-dependent bactericidal mechanism was operating between EG neutrophils and those within *Tg(Runx1:cebpb-CG2;lyz:DsRed2)* larvae, we first investigated the expression of selected mitochondrial respiratory complex subunits and assembly factors that our RNA-seq analysis uncovered were elevated in EG neutrophils following infection. This analysis revealed significantly increased expression for *ndufs3*, *uqcc2*, *ndufs2*, and *ndufaf4* in neutrophils FACS-isolated from infected *Tg(Runx1:cebpb-CG2;lyz:DsRed2)* larvae at 3 hours postinfection (the same time point postinfection used for our RNA-seq analysis), when compared to infected neutrophils similarly isolated from *Tg(lyz:DsRed2)*/WT larvae (Fig. 6, G and H). Also similar to EG neutrophils, the elevated bactericidal activity of neutrophils within *Tg(Runx1:cebpb-CG2;lyz:DsRed2)* larvae was abolished in the presence of MitoTEMPO (Fig. 6I and fig. S7D). In addition, neutrophils from infected *Tg(Runx1:cebpb-CG2;lyz:DsRed2)* larvae had a trend for increased expression of *tfam* and *timm23a* (albeit not to statistical significance), and their mitochondrial mass significantly increased following infection (Fig. 6J and fig. S7E). Collectively, these data strongly suggest that overexpressing *cebpb* in HSPCs is sufficient to enhance granulopoiesis and produce neutrophils with similarly boosted bactericidal activity as those generated during infection-driven EG.

## DISCUSSION

Here, we show that larvae populated with EG neutrophils demonstrate enhanced survival to a secondary lethal infection. We reveal that EG neutrophils can kill intracellular bacteria faster and produce more ROS than SS neutrophils, phenotypes that are maintained following transplantation into infection-naïve hosts. Examining the transcriptomes of EG neutrophils, we uncovered an infection-induced transcriptional program that likely operates within EG neutrophils to elevate mtROS production that we show contributes toward their enhanced bactericidal activity. Last, we provide evidence that the elevated bactericidal activity of EG neutrophils is instructed at the level of infection-experienced HSPCs and that directing *cebpb* expression within infection-naïve HSPCs is sufficient to drive this hematopoietic program and produce neutrophils with similarly boosted bactericidal activity.

The enhanced survival of EG larvae to subsequent infection, as shown here, has been similarly reported when driving EG with *Shigella flexneri*, where the authors also show an expanded HSPC compartment (*19*), suggesting that this host response is not pathogen specific but rather a more heterologous protection mechanism. In this study, we reveal that EG neutrophils have enhanced bactericidal activity that operates in a cell-intrinsic fashion. This appears to provide heterologous protection as EG neutrophils could kill intracellular bacteria that were the same as those used for the initial “priming” challenge (*Salmonella*) or different (*S. iniae*). Although our EG larvae are populated with increased numbers of neutrophils [~2.5-fold, as shown here and (*17*)], we do not believe that this substantially contributes to increased survival in our infection model given that similar numbers of neutrophils were recruited to infection sites within SS and EG larvae. The neutrophil and macrophage ablation experiments performed in this study clearly reveal that EG neutrophils, at least in part through their enhanced bactericidal activity, contribute to the survival advantage observed for EG larvae. Given that EG larvae still maintain a survival advantage in the absence of neutrophils, our results also reveal a non-neutrophil component to this protection. Given that macrophages are well established effectors of innate immune memory (*30*), we suspect that trained macrophages are largely responsible for this residual protection. However, our study shows that macrophages (whether trained or not) are dispensable for the enhanced bactericidal activity of EG neutrophils. Furthermore, given that EG neutrophils transplanted into infection-naïve hosts (where no training occurs) maintain their ability to kill intracellular bacteria faster, we believe that the boosted bactericidal activity of EG neutrophils is a cell-intrinsic feature of these cells. Although we are confident that the vast majority of EG neutrophils examined in our study derive from HSPCs, a limitation of our EG model, when studying SS neutrophils, is that we cannot distinguish between those that are “primitive” (non-HSPC derived) or “definite” (HSPC derived). However, the fact that bactericidal differences between neutrophils derived from transplanted infection-naïve and infection-experienced HSPCs mirrored those observed for SS and EG neutrophils, primitive SS neutrophils are likely similar to those derived from HSPCs, at least with respect to their bactericidal activity.

Our transcriptomic analysis showed that EG neutrophils had elevated expression of genes associated with mitochondrial respiratory chain complex assembly, suggesting enhanced oxidative phosphorylation (OXPHOS) capacity. Notably, the GO term “fatty acid catabolic process” that includes β-oxidation of fatty acids (a process tightly linked to OXPHOS) was one of the top enriched pathways in our transcriptomic analysis. Neutrophils can engage mitochondrial fatty acid oxidation to support effector functions, including ROS production (*54*, *55*). Supporting our finding, a previous study has demonstrated that neutrophils derived from demand-adapted granulopoiesis have enhanced antitumor activity with OXPHOS being similarly identified as a strongly enriched pathway (*14*). OXPHOS generates mtROS largely through electron leakage at ETC complex I and III, and genes involved in the assembly of these complexes were highly represented in our analysis (*33*). We revealed that bacteria-laden EG neutrophils produced significantly more mtROS, but not in the presence of the mitochondrial superoxide scavenger MitoTEMPO. Furthermore, MitoTEMPO-treated EG neutrophils had significantly reduced bacterial killing capacity when compared to untreated EG neutrophils, indicating that production of mtROS contributes to the elevated bactericidal activity of EG neutrophils. Production of mtROS is known to increase in both macrophages and neutrophils following infection and has been shown to be directly involved with killing intracellular bacteria in macrophages (*34*–*37*). More recently, mitochondria have been uncovered as a bactericidal ROS generator in human neutrophils to kill intracellular *Staphylococcus aureus* (*38*). Our work suggests that EG neutrophils adopt an infection-responsive transcriptional program to enhance OXPHOS and boost their bactericidal activity through enhanced mtROS production.

In addition to genes associated with mitochondrial respiratory chain complex assembly, EG neutrophils also up-regulated expression of *tfam* following infection, a core component of the transcriptional machinery in mitochondria that drives mitochondrial gene expression and promotes mitochondrial biogenesis (*39*, *40*). Supporting conservation of function between zebrafish *tfam* with its mammalian ortholog, *tfam*-deficient zebrafish have reduced mitochondrial DNA copy number and demonstrate OXPHOS deficiency (*56*). In addition, we show that EG neutrophils also increase expression of *timm23a* that encodes the zebrafish ortholog of TIMM23, a mitochondrial protein whose expression correlates with mitochondrial content and mtROS production (*41*). Supporting enhanced mitochondrial biogenesis within EG neutrophils, we show that EG neutrophils have greater mitochondrial content following infection when compared to those not exposed to a secondary challenge or to SS neutrophils (irrespective of infection status). Notably, activated T cells have been shown to increase mitochondria size, volume, and number to support elevated mtROS production (*57*). Further studies are necessary to evaluate whether the infection-responsive increase in mitochondrial mass, as described here in EG neutrophils, contributes to their enhanced mtROS and bactericidal activity.

Only recently has evidence emerged that neutrophils contribute to trained immunity. β-glucan–treated mice generate neutrophils with an enhanced antitumor phenotype and show enhanced expression of genes involved in phagocytosis and ROS metabolic pathways (*14*). In another study, neutrophils from Bacillus Calmette-Guérin (BCG)–vaccinated humans displayed a heightened capacity to kill *Candida albicans* and were characterized by enhanced expression of genes involved in activation and degranulation, increased glycolytic rate, and elevated ROS production when stimulated ex vivo (*13*). Our results show that infection-experienced HSPCs are directly involved in producing neutrophils with elevated antibacterial activity in vivo. To better understand the longevity of protection provided by this source of neutrophils, future work will focus on developing techniques to live image neutrophils derived from transplanted infection-experienced HSPCs within juvenile and adult zebrafish.

In addition to establishing epigenetic memory in HSPCs during trained immunity (*8*), C/EBPβ is a transcriptional regulator of EG where it operates within HSPCs to drive proliferation and direct myeloid commitment (*9*–*11*). We show that directing *cebpb* expression to HSPCs is sufficient to stimulate granulopoiesis and generate neutrophils with enhanced bactericidal activity but is not sufficient to increase HSPC numbers. This failure to drive HSPC proliferation may be the result of an inability of our transgenic construct to generate the specific C/EBPβ isoform that induces HSPC proliferation, called liver-enriched inhibitory protein (LIP) (*10*). Analysis of macrophage numbers within *Tg(Runx1:cebpb-CG2;mpeg1:EGFP)* larvae also suggest that the impact of overexpressing *cebpb* within HSPCs skews hematopoiesis largely toward the neutrophil lineage, analogous to the observed changes in blood production seen in our infection-driven EG model described here and previously (*17*). Similar to EG neutrophils, those within *Tg(Runx1:cebpb-CG2;lyz:DsRed2)* larvae enhance expression of mitochondrial respiratory complex subunits and assembly factors associated with mtROS production following infection and have enhanced bactericidal activity through an mtROS-dependent mechanism. Furthermore, like EG neutrophils, those within *Tg(Runx1:cebpb-CG2;lyz:DsRed2)* larvae enhance expression of genes associated with mitochondrial biogenesis following infection and have greater mitochondrial mass. Collectively, these results strongly suggest that a shared mitochondrial mechanism operates within EG neutrophils and those generated from *cebpb*-overexpressing HSPCs that helps explain their enhanced bactericidal activity toward intracellular bacteria. Notably, we did not detect *cebpb* as an up-regulated gene in our transcriptomic analysis of EG neutrophils. This result is consistent with our previous work showing that infection-responsive *cebpb* expression is induced within HSPCs using the same infection-driven EG model (*17*) and aligns with C/EBPβ operating at the level of HSPCs to help establish epigenetic memory during trained immunity (*8*). Performing chromatin immunoprecipitation sequencing on infection-experienced and *cebpb*-overexpressing HSPCs will be necessary to reveal whether the transcriptional changes we uncovered in EG neutrophils are instructed by epigenetic reprogramming at the level of HSPCs. In summary, our work suggests that infection-experienced HSPCs contribute to trained immunity by providing a source of “demand-adapted” neutrophils with enhanced bactericidal activity through mtROS production.

## MATERIALS AND METHODS

### Zebrafish

All zebrafish research was conducted with the approval of the University of Auckland Animal Ethics Committee (approval numbers AEC001911 and AEC22563). Adult zebrafish (*Danio rerio*) were sourced from the University of Auckland’s Zebrafish Facility. The facility was on an automatic 14-hour light/10-hour dark light cycle. Larval zebrafish were generated by natural spawning and raised at 28°C in E3 medium supplemented with 0.003% phenylthiourea (PTU) to inhibit the development of pigmentation. Where appropriate, zebrafish were anesthetized by supplementing E3 with 4% (v/v) tricaine in E3 medium. Wild-type AB zebrafish were obtained from the Zebrafish International Resource Centre. *Tg(lyz:DsRED2*)^nz50^ and *Tg(lyz:EGFP)*^nz117^ neutrophil-specific reporter lines were used in this study, as well as the *Tg(mpeg1:EGFP)^gl22^* and *Tg*(*mpeg1:Gal4FF*)^gl25^/*Tg(UAS-E1b:nfsB-mCherry)*^c264^ lines [herein referred to as *Tg*(*mpeg1:nfsB-mCherry*)] to label macrophages and the *Tg(kdrl:RFP)*^la4^ line to label blood vessels (*23*, *58*–*60*). In addition, the *Tg(mpx:Dendra2)*^nz5^ and *Tg(Mmu.Runx1:EGFP)*^nz6^ [herein referred to as *Tg(Runx1:EGFP)*] reporter lines were recreated in-house from gifted plasmids for use in this study that mark neutrophils and HSPCs, respectively (*25*, *47*). For neutrophil ablation experiments, the *Tg(lyz:YFP-NTR2.0)*^nz53^ line was used (*26*).

### Generation of the *Tg(Mmu.Runx1:cebpb-CG2)* reporter line

The *Tg(Mmu.Runx1:cebpb-CG2)* line [herein referred to as *Tg(Runx1:cebpb-CG2)*] was generated via Gateway cloning (Invitrogen). The zebrafish ortholog of *C/EBP*β (*cebpb*) was ordered as a gBlock Gene Fragment from Integrated DNA Technologies. This included the 840-bp sequence (stop codon removed) of the single *cebpb* exon (ENSDARG00000042725) with an additional 4-bp sequence (CACC) at the 5′ end of the gBlock to facilitate directional cloning. This sequence was cloned into the pENTR/D-TOPO vector as per the manufacturer’s protocol (Invitrogen) to produce the pME-*cebpb* plasmid. pME-*cebpb*, p5E-*Mmu.Runx1* [mouse *Runx1* +23 enhancer, gift from L. Zon (*49*)], and p3E-*p2a-EGFP* (a gift from Omid Delfi) were cloned into the pDestTol2CG2 destination vector (*53*) via LR clonase II plus recombination, as per the manufacturer’s protocol (Invitrogen). pDESTTol2CG2 contains the heart-marking *cmlc2:EGFP* transgenesis marker. One nanoliter of this construct was injected into single-cell stage AB embryos with the following injection mix: 1 μl (200 ng) of plasmid DNA, 1 μl (250 ng) of transposase mRNA, 3 μl of phenol red, and 5 μl of ultrapure water. At 24 hours postinjection, embryos were selected for the green heart marker, raised to sexual maturity and outcrossed to AB to identify germline transmitting founders. Positive F1 progeny was then raised as a stable transgenic line.

### Bacterial injections

GFP-expressing *S. enterica* (herein referred to as *Sal*-GFP) was prepared by inoculating 4 ml of LB broth supplemented with kanamycin (25 μg/ml) and culturing overnight at 28°C. This was diluted 1:10 in equal parts LB and Dulbecco’s modified Eagle’s medium. This subculture was then incubated at 37°C for 45 min while shaking at 200 revolutions per min (rpm). The bacterial pellet was obtained by centrifugation and resuspended with sterile PBS and 0.25% phenol red to the desired final concentration. *S. iniae* was prepared by inoculating 2 ml of Todd Hewitt broth with 2% (w/v) yeast and 20% (w/v) peptone (THY + P) supplemented with kanamycin (50 μg/ml) and cultured for 24 hours at 37°C. This was diluted 1:10 in THY + P broth and incubated at 37°C without shaking until the optical density at 600 nm was 0.4. One milliliter of the culture was centrifuged and resuspended in 1 ml of THY + P broth supplemented with 25% glycerol and stored at −80°C until required. Immediately before injecting, frozen stocks were thawed, centrifuged, and resuspended in sterile PBS containing 0.25% phenol red to the desired final concentration.

Larvae were raised to the appropriate developmental time point, anesthetized in 4% tricaine, and then arrayed laterally in 3% (w/v) methylcellulose in E3 medium. Approximately 1 nl of the bacterial injection mix was microinjected into the appropriate anatomical location (hindbrain ventricle, circulation, or somite), as previously described (*17*). The dose of bacteria was validated for each experiment by diluting an injection bolus 1:10 and 1:100 in sterile PBS, both before and after the infections, and plating on LB agar (*Sal*-GFP) or THY + P agar (*S. iniae*) supplemented with kanamycin.

### Survival analysis

Survival analysis was performed as previously described (*36*). Larvae were monitored for up to 5 days following infection and dead larvae, as judged by cardiac arrest, were removed, and counted at 24-hour intervals.

### Bacterial CFU enumeration

Bacterial CFUs within individual larvae were performed as previously described (*36*). Briefly, larvae were rinsed in E3 medium at set time points following infection and placed into separate microtubes containing 100 μl of sterile PBS supplemented with 1% Triton X-100. Each larva was homogenized with a sterile pestle, and the homogenate was diluted in a 100-fold dilution series. Ten microliters of each dilution (max 1:10^8^) was spot plated in triplicate onto LB agar supplemented with kanamycin (25 μg/ml). Plates were incubated at 28°C overnight. Bacterial burdens were determined by calculating the average number of colonies recovered at the highest dilution that grew colonies in each replicate. Bacterial burdens were quantified for 8 to 15 larvae per time point.

### Confocal microscopy

Larvae were live imaged on an Olympus Fluoview FV1000 laser scanning confocal microscope equipped with an incubation chamber set to 29°C. Larvae were anesthetized with 4% tricaine and mounted in 0.7 to 1% (w/v) low melting point agarose in E3 medium supplemented with 0.003% PTU and 3.2% tricaine. The lower (0.7%) concentration of agarose was used for longer-term time-lapse imaging. Images were captured with a 60× water immersion lens, a scan format of 512 × 512 pixels and a zoom setting of 1.5 to 2.5×. All imaging parameters were kept identical within, and across, imaging experiments when measuring neutrophil killing rates and quantifying CellROX and MitoSOX fluorescence within bacteria-laden neutrophils, including laser voltage, resolution, scanning speed, offset, and gain.

### Macrophage ablation

For liposome clodronate–mediated macrophage ablation, liposomes were injected into both the hindbrain ventricle and circulation as previously described (*29*). Notably, given the requirement of blood circulation for recruitment of innate immune cells to the hindbrain infection site, only liposome-injected larvae with unaffected blood flow were used for subsequent experiments.

### Neutrophil ablation

For neutrophil ablation experiments, *Tg(lyz:YFP-NTR2.0)* larvae were given a 0.2 mM dose of mtz (by immersion) at 4 dpf (2 dpi) to effect complete neutrophil ablation within 6 hours. This was followed by continuous exposure to 0.1 mM mtz following infection for survival analyses.

### Immunofluorescence

Immunofluorescent detection of neutrophils within *Tg(lyz:DsRED2)* larvae was performed as previously described (*36*). The primary and secondary antibodies used for the detection of DsRED2 their dilutions were rabbit anti-DsRED2, 1:400 (Takara Bio, catalog no. 632496) and goat anti-rabbit Alexa Fluor 568, 1:400 (Invitrogen, A-11011).

### Quantifying neutrophil killing rates, ROS, and mtROS production

Neutrophil bacterial killing rates were measured at 2 to 4 hours postinfection by time-lapse confocal microscopy as previously described (*24*). Z-stacks (1.5-μm step size) of individual bacteria-laden neutrophils were collected for 8 to 15 min. Imaged neutrophils had to satisfy the following criteria to be included in a dataset: There was no evidence of additional bacterial phagocytosis over the imaging period; the neutrophil needed to remain within the *X*-, *Y*- and *Z*-dimensions throughout the imaging period; and the imaging period needed to be between 8 and 15 min, which was sufficient to detect a killing rate while minimizing photobleaching. Time-lapse movies were analyzed using Volocity v6.3 image analysis software. Within bacteria-laden neutrophils that satisfied the above criteria, the volume (μm^3^) of GFP-labeled bacteria was measured using the volume measurement tool in Volocity at the beginning and end of each time-lapse movie. First, objects were identified using GFP fluorescence signal intensity (using a constant threshold setting), a region of interest (ROI) was manually positioned around the neutrophil in *X*-, *Y*-, and Z-dimensions, and the sum of the volumes for all objects within the ROI was quantified to give a total volume. From this neutrophil, killing rates were calculated as (initial volume − final volume)/time, or Δ μm^3^/min, where a positive value is interpreted as intracellular bacterial killing and a negative value as bacterial growth.

CellROX Deep Red Reagent, a cell-permeable probe that fluoresces following oxidation by ROS, was used to measure ROS production in neutrophils as previously described (*24*). Briefly, the bacterial injection mix was supplemented with 50 μM CellROX and delivered into the hindbrain ventricle by microinjection. Z-stacks (1.5-μm step size) of bacteria-laden neutrophils were then imaged by confocal microscopy at 2 to 4 hours postinfection. The total fluorescence intensity of CellROX within neutrophils containing intracellular bacteria was measured using Volocity v6.3. *Z*-stacks were first manually inspected to select for neutrophils containing bacteria. Next, a ROI was drawn around individual bacteria-laden neutrophils in the *X*-, *Y*-, and *Z*-dimensions. Using the threshold function (using a constant threshold setting) for the CellROX channel (excitation/emission maxima of 640/665 nm), the sum of the greyscale intensity values of individual voxels within the ROI was measured and plotted as the total fluorescence intensity CellROX/neutrophil.

MitoSOX Red (excitation/emission maxima of 396/610 nm), a cell-permeable fluorescent probe selective for mitochondria-specific superoxide, was used to measure mtROS in neutrophils. To quantify mtROS, we adapted a protocol we previously used to measure mtROS within larval zebrafish macrophages using MitoSOX (*36*). Briefly, the bacterial injection mix was supplemented with 50 μM MitoSOX. Live confocal imaging was used to take single Z-stacks (1.5-μm step size) of bacteria-laden neutrophils from 2 to 4hours postinfection. The fluorescence intensity of MitoSOX within neutrophils containing intracellular bacteria was measured using ImageJ/Fiji. Z-stacks were first manually inspected to select for neutrophils containing bacteria. Next, a ROI was drawn around individual bacteria-laden neutrophils in the *X*-, *Y*-, and *Z*-dimensions, and the controlled total cell fluorescence (CTCF) of MitoSOX/neutrophil was determined using ImageJ/Fiji as previously described (*61*). CTCF was used for this probe to correct for background noise.

### Chemical treatments

MitoTEMPO, a mitochondrial superoxide scavenger, was injected immediately following bacterial infection into the hindbrain ventricle at a concentration of 250 μM in dimethyl sulfoxide (DMSO) as previously described (*29*).

### Photoconversion

*Tg(mpx:Dendra2)* larvae were viewed under a 20× water dipping objective lens. A ROI was selected using the FluoView FV1000 software around the Dendra2-expressing cells of interest in the CHT, and a 405-nm laser was targeted to the ROI for 1 min to photoconvert Dendra2 from green (excitation/emission maxima of 490/507 nm) to red (excitation/emission maxima of 553/573 nm) fluorescence.

### Live fluorescence microscopy

Larvae were anesthetized with 4% tricaine and arrayed in 3% methylcellulose in E3. Images were taken using a DS-U2/L2 camera fitted to a Nikon SMZ1500 fluorescence stereomicroscope.

### Flow cytometry and FACS

Larvae were dissociated for flow cytometry/FACS as previously described with modifications (*62*). Briefly, dechorionated larvae were rinsed in calcium-free Ringer’s solution supplemented with 2 mM MgCl_2_ and 10 mM D+ glucose on ice for 15 min. Larvae were de-yolked by repeated passage through a 200-μl pipette tip, and the supernatant was discarded. Larvae were digested in 0.25% trypsin-EDTA in PBS for 1 to 1.5 hours at 28°C with manual agitation every 10 min. The digestion was inhibited by adding CaCl_2_ to a final concentration of 1 mM and 5% fetal bovine serum (FBS). The dissociated material was centrifuged at 260*g* for 5 min at 4°C, and the supernatant was removed by decanting. The cell pellet was resuspended in ice-cold PBS supplemented with 5% FBS. Cells were passed through a 40-μm cell strainer (BD Falcon) and stored on ice before flow cytometry or FACS. Flow cytometry was performed on a BD LSR II flow cytometer, and cells were gated on the basis of forward and side scatter characteristics and GFP, DsRED2, yellow fluorescent protein (YFP), or mCherry expression. Cells were FACS-isolated on a BD FACS Aria II into PBS supplemented with 5% FBS and either stored on ice prior for transplantation or processed for expression analysis by quantitative polymerase chain reaction (qPCR) or RNA-seq.

### MitoTracker staining and quantification

Neutrophil mitochondrial mass was quantified using the MitoTracker Deep RED FM probe (Thermo Fisher Scientific) as per the manufacturer’s recommendations, with the following modifications. Larvae were first dissociated, as above, before cells were transferred into 1 ml of PBS supplemented with 10 mM Hepes and 1 mM EDTA. MitoTracker Deep RED FM probe (excitation/emission maxima of 644/665 nm) was added to a final concentration of 300 nM followed by incubated at 28°C for 30 min. Following centrifugation, stained cells were then fixed in 4% paraformaldehyde for 10 min, centrifuged, and resuspended in PBS supplemented with 10 mM Hepes and 1 mM EDTA. Fluorescence intensity measurements of MitoTracker Deep RED FM (relative fluorescence intensity in arbitrary units) were then measured using a BD FACS Aria II (640-nm laser and 670/30 bandpass filter) on gated DsRED2-positive cells (552-nm laser and 585/15 bandpass filter).

### Cytospin preparation and cell staining

Cells were collected via FACS as described above. Samples were spun at 200*g* for 5 min at 4°C. The supernatant was discarded, and the cell pellet was resuspended in 250 μl of cytospin buffer [3 mM EDTA, 2% bovine serum albumin (BSA), and 1% FBS in 1× PBS]. Cells were then fixed to slides using an Aerospray Hematology Pro cytospin and stained immediately (Wright-Giemsa) using the in-built slide staining mechanism.

### Cell transplantation

Neutrophils or HSPCs were isolated from donor larvae and transplanted into recipient larvae as previously described with modifications (*32*). Briefly, around 5000 cells were FACS-isolated into PBS supplemented with 5% FBS and then centrifuged at 260*g* for 10 min at 4°C. The supernatant was removed by pipetting leaving ~20 μl behind to resuspend the pellet. Recipient larvae were anesthetized with 4% tricaine and arrayed in 3% methylcellulose in E3 medium. Three microliters of the cell resuspension was loaded into a microinjection needle, and an MPPO-2 Pressure Injector (Applied Scientific Instrumentation) was used to inject cells into the appropriate location within recipient larvae (hindbrain ventricle for neutrophils, circulation for HSPCs). Transplanted larvae were returned to fresh E3 with 0.003% PTU and monitored for successful engraftment using confocal or fluorescent microscopy from 1 day posttransplant.

### RNA extraction and qPCR

Total RNA was extracted from FACS-isolated sorted cells using the RNeasy Micro Plus Kit (Qiagen) where cells were directly sorted into 350 μl of RLT Plus buffer and RNA extracted as per the manufacturer’s protocol. Total RNA was reverse transcribed into cDNA using the iScript cDNA Synthesis Kit (Bio-Rad), and qPCR was performed using the iTaq Universal SYBR Green Supermix (Bio-Rad) and the QuantStudio 6 K Flex Real-Time PCR System (Life Technologies, Thermo Fisher Scientific). The following primer pairs were used for *runx1*, forward 5′-AATGACCTGCGTTTCGTGGG-3′, reverse 5′-TGTCGGTGGCGTCGTGG-3′; *cmyb*, forward 5′-ACAACAGGCACTACCAATCTCC-3′, reverse 5′-CAATGCCAACCGAACTGTCC-3′; *cebpb*, forward 5′-CTTTCCACAGCACTAACGCC-3′, reverse 5′-AGTCTATGGCTTTCTCGTGC-3′; *ndufs3*, forward 5′-TGGTATGAGAGAGAGGTTTGG-3′, reverse 5′-CGGGAAATCTTTCCTGAATGG-3′; *uqcc2*, forward 5′-CGATTTCTGAAGCTGTGCGAG-3′, reverse 5′-TTTCTCTGGGTCTGAAATCTGTG-3′; *ndufs2*, forward 5′-AACATTCAGGCTCCACCTCG-3′, reverse 5′-ATGGCTCCGATGTCAAGGG-3′; *ndufaf4*, forward 5′-CAGTCGCTCAGGGTGATGC-3′, reverse 5′-GGTTCTTGTGATTGTTCAGGGC-3′; *tfam*, forward 5′-TGCATCTGTTGTGAGGTGTTC-3′, reverse 5′-TGACGGTGGGCTGCATATC-3′; *timm23a*, forward 5′-TTTCAGGAGTACCGTTGACTGG-3′, reverse 5′-CAGCTCAAAGCGACCACGAG-3′; *ef1a*, forward 5′-TGCCTTCGTCCCAATTTCAG-3′, reverse 5′-TACCCTCCTTGCGCTCAATC-3′. Each qPCR experiment was performed in biological and technical triplicate. Expression levels were normalized to *ef1*α and calculated using the ΔΔCt method.

### RNA-seq and data analysis

Larvae were prepared for FACS as described above, with a change in protocol that the cells were sorted into 0.1% BSA in PBS instead of 5% FBS to prevent inhibition of downstream enzymatic reactions. One thousand cells per sample were sorted directly into a “lo-bind” 1.5-ml Eppendorf tube containing 1 μl of 10× lysis buffer, 1 μl of ribonuclease inhibitor, and 5 μl of nuclease-free water. Nuclease-free water was then added to 10.5 μl. Sorted cells were then vortexed briefly to homogenize and lyse the cells, briefly centrifuged, and then stored at −80°C. Frozen samples were thawed on ice before cDNA synthesis as per the SMART-Seq v4 Ultra Low Input RNA kit for sequencing (Takara Bio) manufacturer’s protocol. cDNA from each sample was run on an Agilent 2100 Bioanalyzer to measure cDNA fragment size (in base pairs), concentration, and purity. One nanogram total cDNA was the input for each sample into the Nextera XT DNA Library Preparation Kit (Illumina). DNA libraries were prepared as per the manufacturer’s protocol. Libraries were checked for size distribution, purity, and concentration on an Agilent 2100 Bioanalyzer. Libraries were sequenced using NextSeq 500 in single-end reads, 75-bp read length.

Single-end reads were checked for quality using FastQC, and filtered, cleaned reads were mapped to the zebrafish reference genome (GRCz11) from Ensembl using HISAT2. Expression levels were measured as fragments per kilobase of transcript per million mapped reads using the Cuffquant and Cuffnorm components of Cufflinks software. DESeq was then used to analyze the DEGs between sets of two groups as a control-treatment pairwise comparison. The expression fold change (log_2_FC) was set to >1, and the false discovery rate was set to <0.05 as screening criteria for DEGs. DEGs were functionally annotated using GO enrichment analysis. Analysis of RNA-seq data was performed with assistance from CD genomics (https://cd-genomics.com/).

### Statistical analysis

Statistical significance was determined using GraphPad Prism 8. Multiple comparison analysis was carried out by ordinary one-way analysis of variance (ANOVA) with Tukey’s multiple comparisons test. Pair-wise comparisons were carried out by Student’s unpaired *t* test. Kaplan-Meier survival plot analysis was carried out by the Gehan-Breslow-Wilcoxon method. Statistical significance was defined by *P* values < 0.05. The statistical test used and *P* values are indicated in each figure legend.
