# Supplementary material for: Infection-experienced HSPCs protect against infections by generating neutrophils with enhanced mitochondrial bactericidal activity
Source: Sci Adv. 2023 Sep 6;9(36):eadf9904. doi: 10.1126/sciadv.adf9904 (PMC10482338; doi:10.1126/sciadv.adf9904)
Supplement: Supplementary file 1 — Figs. S1 to S7 [file sciadv.adf9904_sm.pdf]

Science Advances
AAAS

Supplementary Materials for

Infection-experienced HSPCs protect against infections by generating neutrophils with enhanced mitochondrial bactericidal activity

Hannah Darroch *et al.*

Corresponding author: Christopher J. Hall, c.hall@auckland.ac.nz

Sci. Adv. **9**, eadf9904 (2023)
DOI: 10.1126/sciadv.adf9904

This PDF file includes:

Figs. S1 to S7

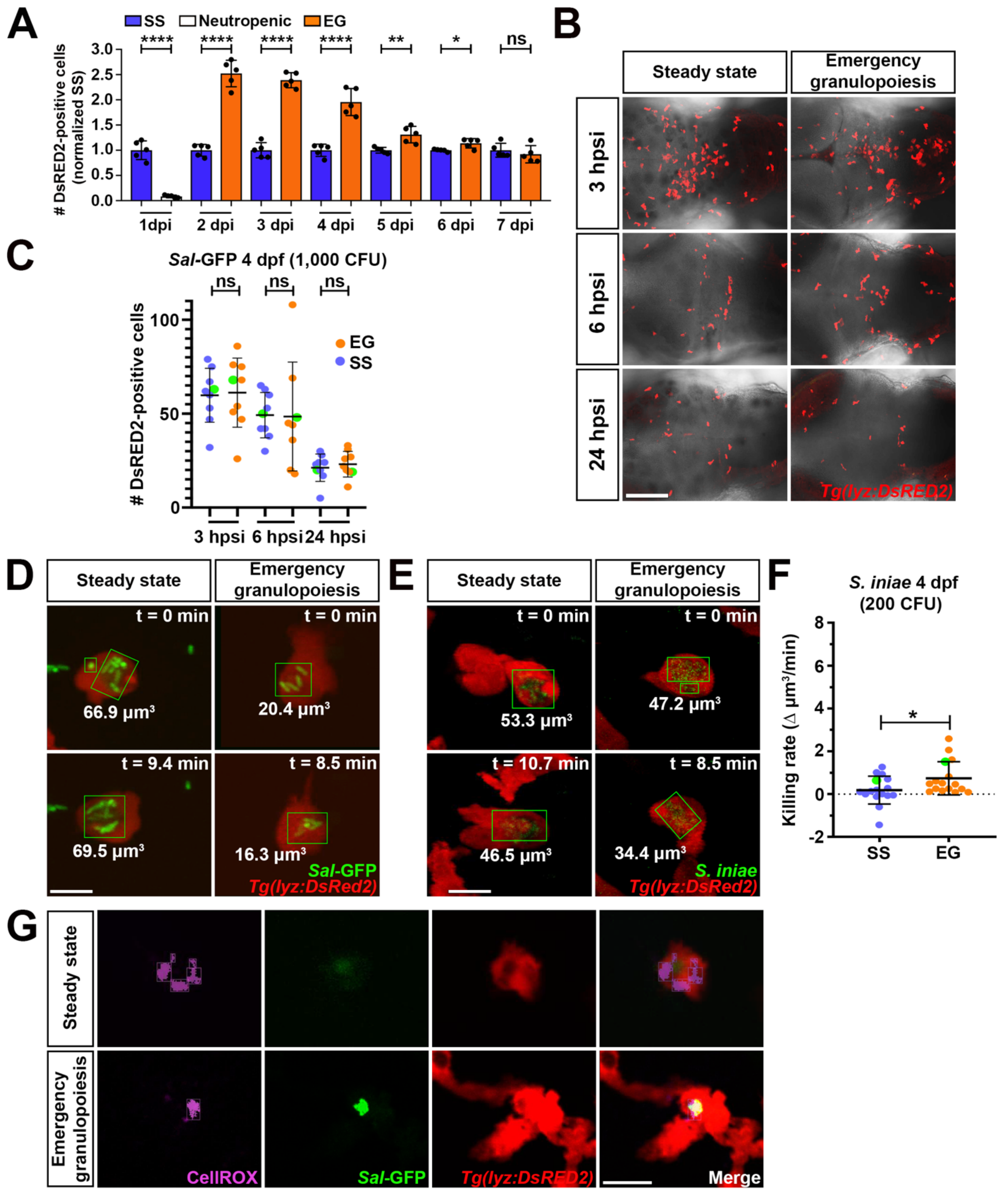

Fig. S1. Neutrophil numbers in EG larvae return to SS levels by 7 dpi, similar numbers of neutrophils are recruited to infections within SS and EG larvae and live confocal imaging of neutrophils following *Sal*-GFP and *S. iniae* infection. (**A**) Flow quantification of neutrophils from *Tg(lyz:DsRED2)* EG and SS larvae (as selected for in Fig. 1A-D) 1, 2, 3, 4, 5, 6 and 7 days post injection (dpi) with *Sal*-GFP and PBS at 2 dpi (n=10-15 larvae/sample, 5 experimental replicates). (**B**) Immunofluorescence detection of neutrophils in the hindbrain ventricles of SS and

EG *Tg(lyz:DsRED2)* larvae at 3, 6, and 24 hours post secondary injection (hpsi) with *Sal*-GFP. (**C**) Quantification of neutrophils as detected in **B**. Green data points highlight larvae shown in **B**. (**D**) Frame shots from live time-lapse confocal imaging of SS and EG neutrophils within *Tg(lyz:DsRED2)* larvae showing volumes of intracellular *Sal*-GFP at the beginning (t=0) and end of the time-lapse experiment. (**E**) Frame shots from live time-lapse confocal imaging of SS and EG neutrophils within *Tg(lyz:DsRED2)* larvae showing volumes of intracellular *S. iniae* at the beginning (t=0) and end of the time-lapse experiment. (**F**) Bacterial killing rates of SS and EG neutrophils following *S. iniae* infection. Green data points highlight killing rates of neutrophils as shown in **E**. (**G**) Live confocal imaging of ROS production within individual *Sal*-GFP-laden SS and EG neutrophils within *Tg(lyz:DsRED2)* larvae, as detected by CellROX fluorescence. Error bars, mean ± SD; ns, not significant, * $P<0.05$, ** $P<0.01$, **** $P<0.0001$; unpaired Student's t-test (**A**, **C** and **F**). CFU, colony-forming units. Scale bars, 100 μm in **B** and 10 μm in **D**, **E** and **G**.

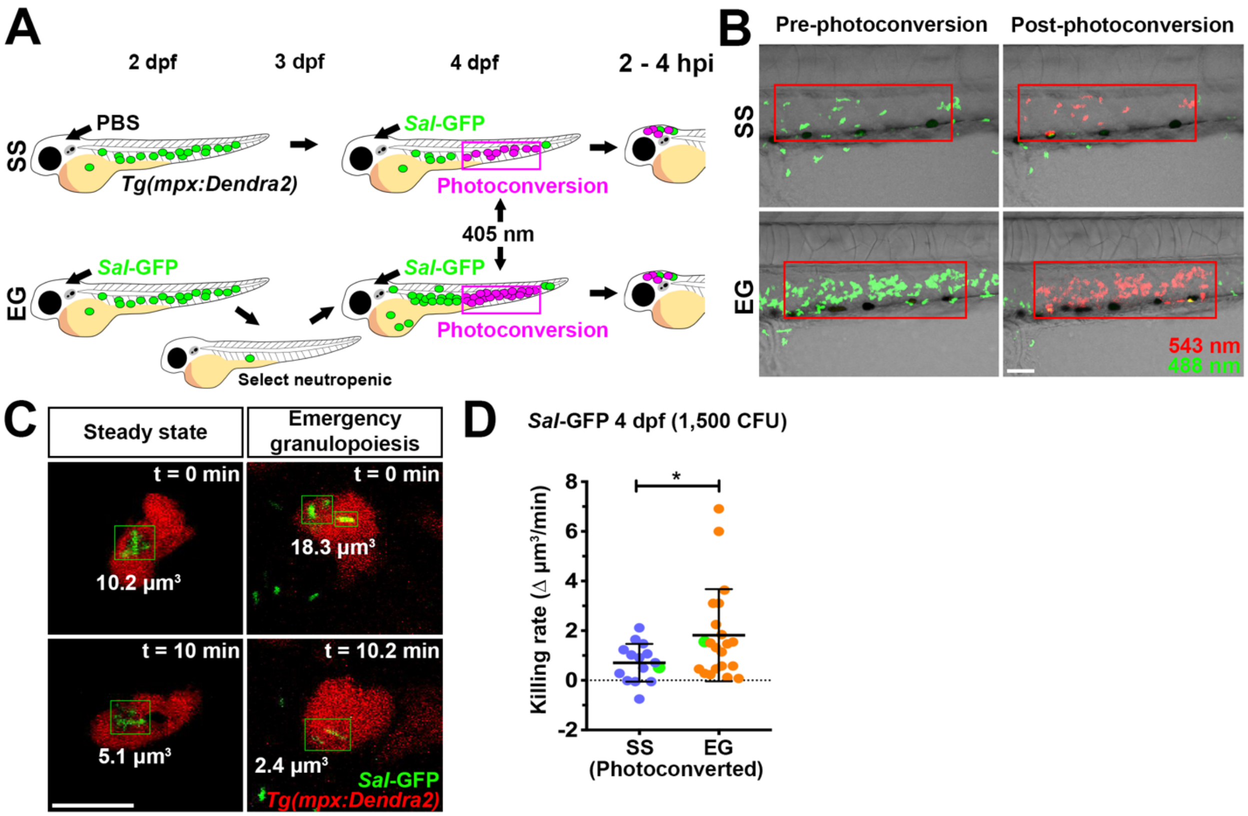

Fig. S2. Enhancing for the selection of EG neutrophils through photoconversion of newly generated neutrophils in the CHT. (**A**) Schematic illustrating strategy to enhance selection of EG neutrophils by photoconverting neutrophils within the CHT region. (**B**) Live imaging of SS and EG neutrophils within the CHT regions of *Tg(mpx:Dendra2)* larvae immediately prior to, and following, photoconversion. Red box marks photoconverted region. (**C**) Frame shots from live time-lapse confocal imaging of photoconverted SS and EG neutrophils within *Tg(mpx:Dendra2)* larvae showing volumes of intracellular *Sal*-GFP at the beginning (t=0) and end of the time-lapse experiment. (**D**) Bacterial killing rates of photoconverted SS and EG neutrophils following *Sal*-GFP infection. Green data points highlight killing rates of neutrophils as shown in **C**. Error bars, mean ± SD; * *P* <0.05; unpaired Student's t-test (**D**). CFU, colony-forming units. Scale bars, 50 µm in **B** and 10 µm in **C**.

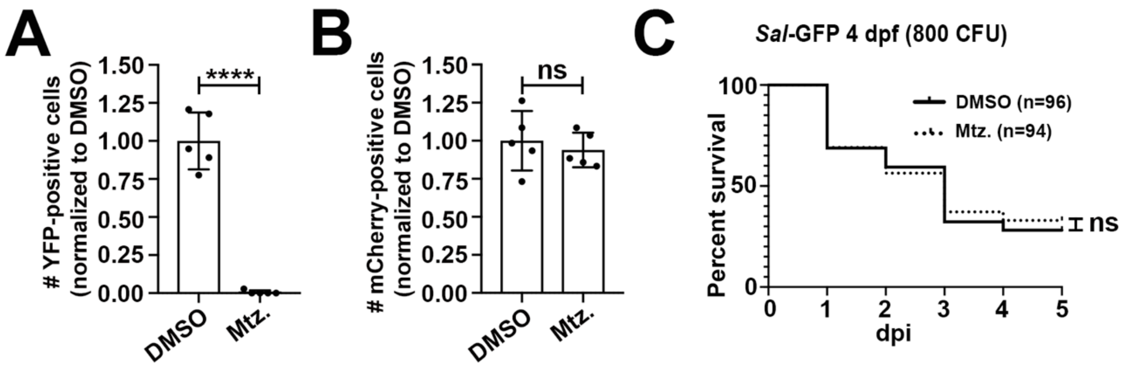

Fig. S3. The metronidazole treatment regimen used in this study has no impact on macrophages within *Tg(mpeg1:nfsB-mCherry)* larvae. (**A**) Flow quantification of YFP-expressing neutrophils from whole *Tg(lyz:YFP-NTR2.0;mpeg1:nfsB-mCherry)* larvae, 5 days following continuous 0.1 mM metronidazole (mtz) treatment, compared to DMSO-treated controls (n=15 larvae/sample, 5 experimental replicates). (**B**) Flow quantification of mCherry-expressing macrophages from whole *Tg(lyz:YFP-NTR2.0;mpeg1:nfsB-mCherry)* larvae, 5 days following continuous 0.1 mM mtz treatment, compared to DMSO-treated controls (n=15 larvae/sample, 5 experimental replicates). (**C**) Kaplan-Meier graph showing survival of DMSO- and 0.1 mM mtz-treated *Tg(mpeg1:nfsB-mCherry)* larvae over 5 days following infection with *Sal*-GFP at 4 dpf. Error bars, mean ± SD; ns, not significant, **** P <0.0001; unpaired Student's t-test (**A** and **B**), Gehan-Breslow-Wilcoxon test (**C**). CFU, colony-forming units.

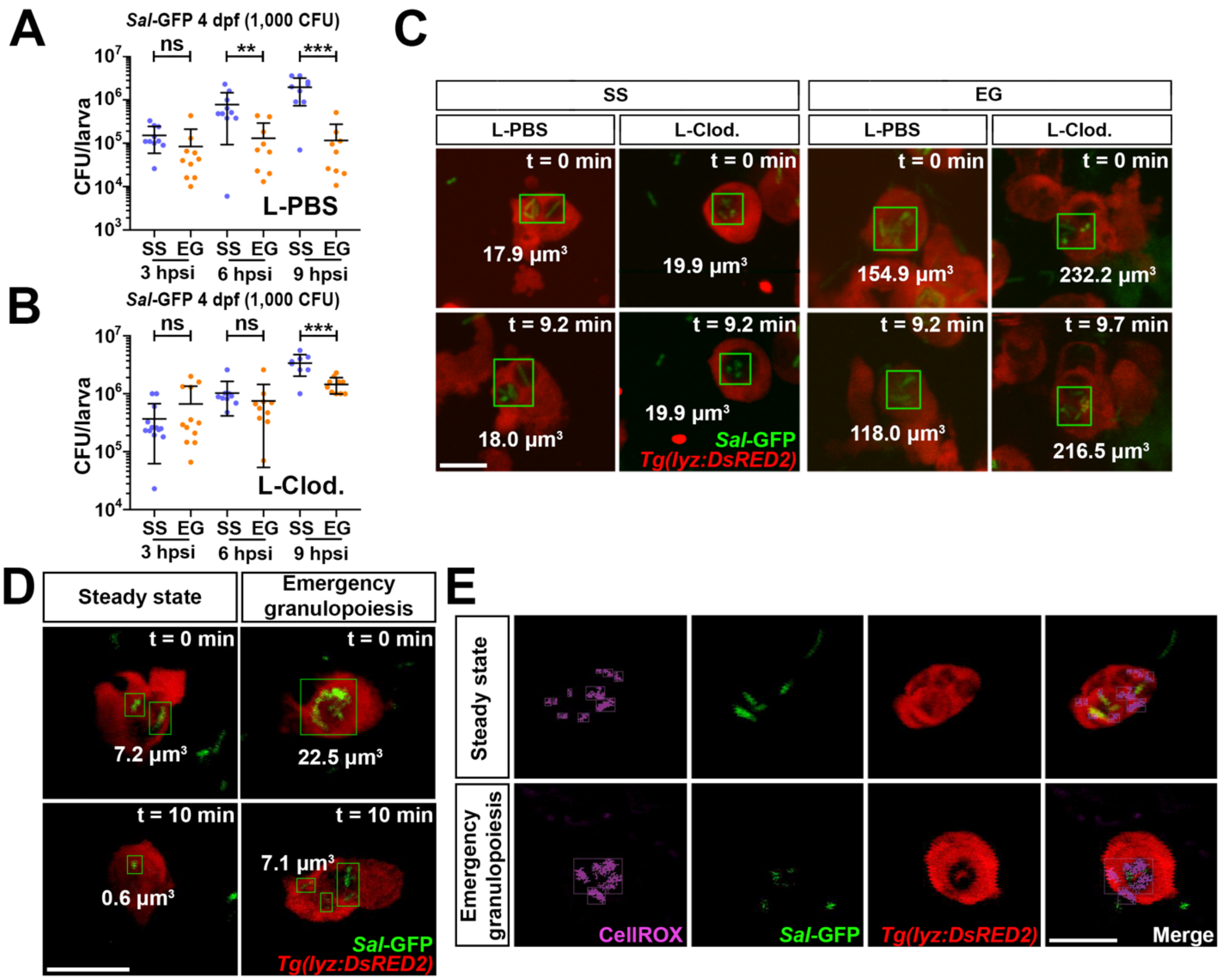

Fig. S4. Bacterial burdens in SS and EG larvae in the presence and absence of macrophages, confocal imaging of neutrophils in the absence of macrophages and transplanted neutrophils following *Sal*-GFP infection. (**A**) Bacterial burdens within individual L-PBS-injected SS and EG larvae at 3, 6 and 9 hours post secondary injection (hpsi) with *Sal*-GFP at 4 dpf. (**B**) Bacterial burdens within individual L-Clod.-injected SS and EG larvae at 3, 6 and 9 hpsi with *Sal*-GFP at 4 dpf. (**C**) Frame shots from live time-lapse confocal imaging of SS and EG neutrophils within L-PBS- and L-Clod.-injected *Tg(lyz:DsRED2;mpeg1:EGFP)* larvae, showing volumes of intracellular *Sal*-GFP at the beginning (t=0) and end of the time-lapse experiment. (**D**) Frame shots from live time-lapse confocal imaging of SS and EG neutrophils transplanted from *Tg(lyz:DsRED2)* larvae into infection-naïve recipient larvae, showing volumes of intracellular *Sal*-GFP at the beginning (t=0) and end of the time-lapse experiment. (**E**) Live confocal imaging of ROS production within individual *Sal*-GFP-laden SS and EG neutrophils transplanted from *Tg(lyz:DsRED2)* larvae into infection-naïve recipient larvae, as detected by CellROX fluorescence. Error bars, mean ± SD; ns, not significant, ** P <0.01, *** P <0.001; unpaired Student's t-test (**A** and **B**). CFU, colony-forming units. Scale bar 10 µm.

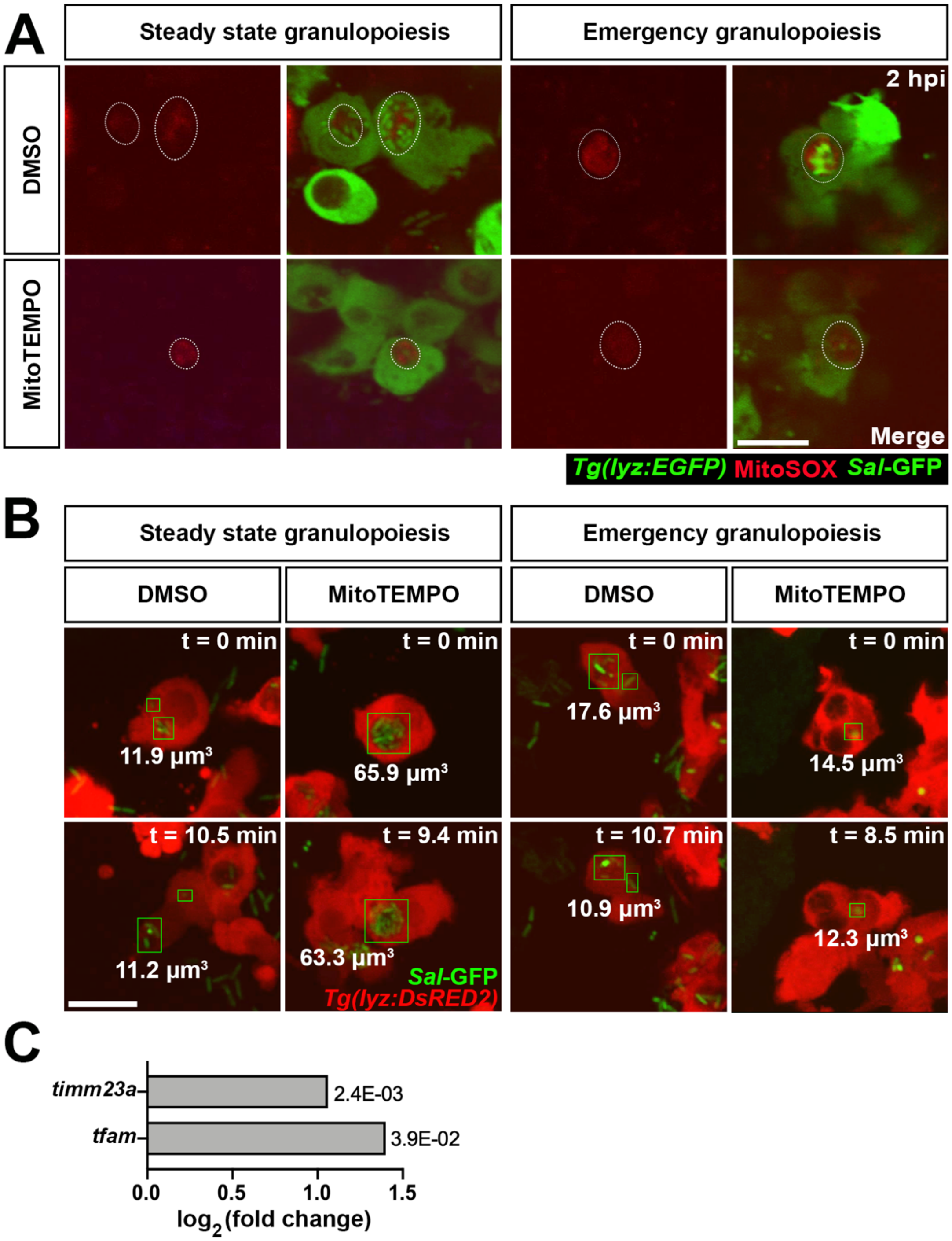

Fig. S5. Confocal imaging of mtROS production within neutrophils, *Sal*-GFP-laden neutrophils following MitoTEMPO treatment and expression of DEGs of interest *timm23a* and *tfam*. (**A**) Live confocal imaging of mtROS production within individual *Sal*-GFP-laden SS and EG neutrophils within *Tg(lyz:EGFP)* larvae*,* as detected by MitoSOX fluorescence, in the presence of DMSO (control) and MitoTEMPO. White dashed lines outline MitoSOX fluorescence within neutrophils. (**B**) Frame shots from live time-lapse confocal imaging of SS and EG neutrophils within *Tg(lyz:DsRED2)* larvae, in the presence of DMSO (control) and MitoTEMPO, showing volumes of intracellular *Sal*-GFP at the beginning (t=0) and end of the time-lapse

experiment. (**C**) Log_2 fold change for DEGs of interest *timm23a* and *tfam* (as described in Fig. 4B), with adjusted *P* values. Scale bar 10 µm.

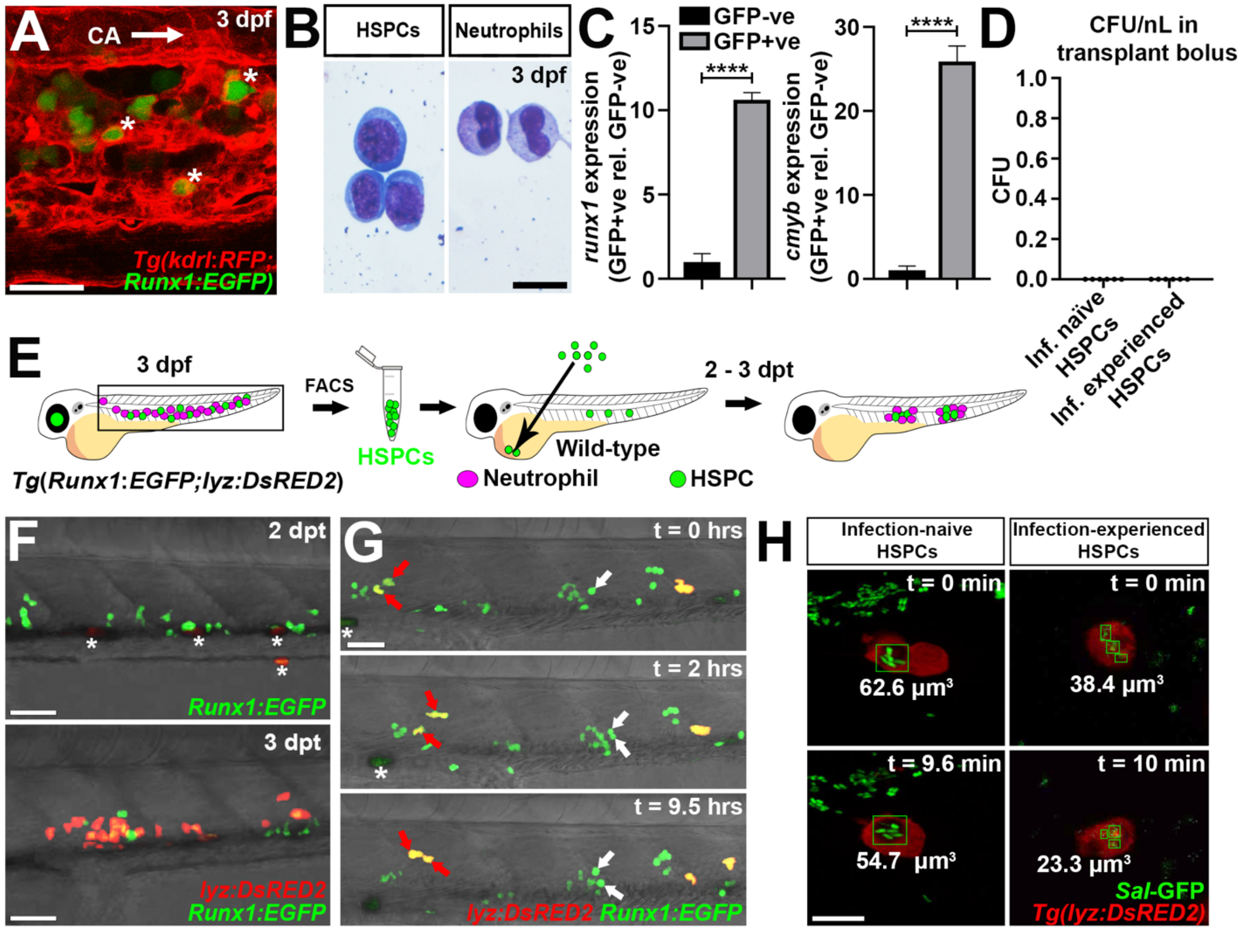

Fig. S6. The *Tg(Runx1:EGFP)* reporter line marks HSPCs, transplanted HSPCs are capable of cell division and contributing to the neutrophil lineage and confocal imaging of neutrophils from infection-naïve and -experienced HSPCs. (**A**) Live confocal imaging of HSPCs within 3 dpf *Tg(Runx1:EGFP;kdrl:RFP)* larvae showing endothelial cells 'cuddling' HSPCs (marked by white asterisks) in the CHT. Arrow marks the caudal artery (CA). (**B**) Histological examination of HSPCs and neutrophils (Wright-Giemsa stained) FACS-isolated from the trunks of 3 dpf *Tg(Runx1:EGFP;lyz:DsRED2)* larvae. (**C**) Expression of *runx1* and *cmyb* within GFP +ve cells (relative to GFP -ve cells) FACS-isolated from the dissected trunks of 3 dpf *Tg(Runx1:EGFP)* larvae, as detected by qPCR (in biological triplicate). (**D**) CFU enumeration of *Sal*-GFP within transplantation boluses (n=6 experiments). (**E**) Schematic illustrating HSPC transplantation protocol. EGFP-expressing HSPCs were FACS-isolated from the dissected trunks of 3 dpf *Tg(Runx1:EGFP;lyz:DsRED2)* larvae and transplanted into the circulation of 2 dpf WT recipient larvae. Transplanted larvae were visually inspected at 1 dpt for HSPC engraftment and DsRED2-expressing neutrophils (derived from transplanted HSPCs) were detectable from 2–3 days post transplant (dpt). In a typical transplantation experiment, 77.0% (SD ± 1.25, n=2 experiments) of injected recipient larvae showed engrafted HSPCs in the circulation/AGM/CHT at 1 dpt. Of HSPC-engrafted larvae, 46.1% (SD ± 0.25, n=2 experiments) possessed *DsRED2*-expressing neutrophils by 2 dpt, and 77% (SD ± 2.05, n=2 experiments) by 3 dpt. (**F**) Live confocal imaging of HSPC-engrafted larva at 2 and 3 dpt. (**G**) Frame shots from live confocal time-lapse of HSPC-engrafted larvae at 2 dpt demonstrating HSPC cell division (white arrows) and transplanted cells progressively expressing *DsRED2* (red arrows). White asterisks mark autofluorescent pigment.

(**H**) Frame shots from live time-lapse confocal imaging of neutrophils derived from infection-naïve and -experienced HSPCs transplanted from *Tg(Runx1:EGFP;lyz:DsRED2)* larvae into WT recipients, showing volumes of intracellular *Sal*-GFP at the beginning (t=0) and end of the time-lapse experiment. Error bars, mean ± SD; **** *P* <0.0001; unpaired Student's t-test (**C**). CFU, colony-forming units. Scale bars, 50 μm in **A**, **F** and **G**, 10 μm in **B** and **H**.

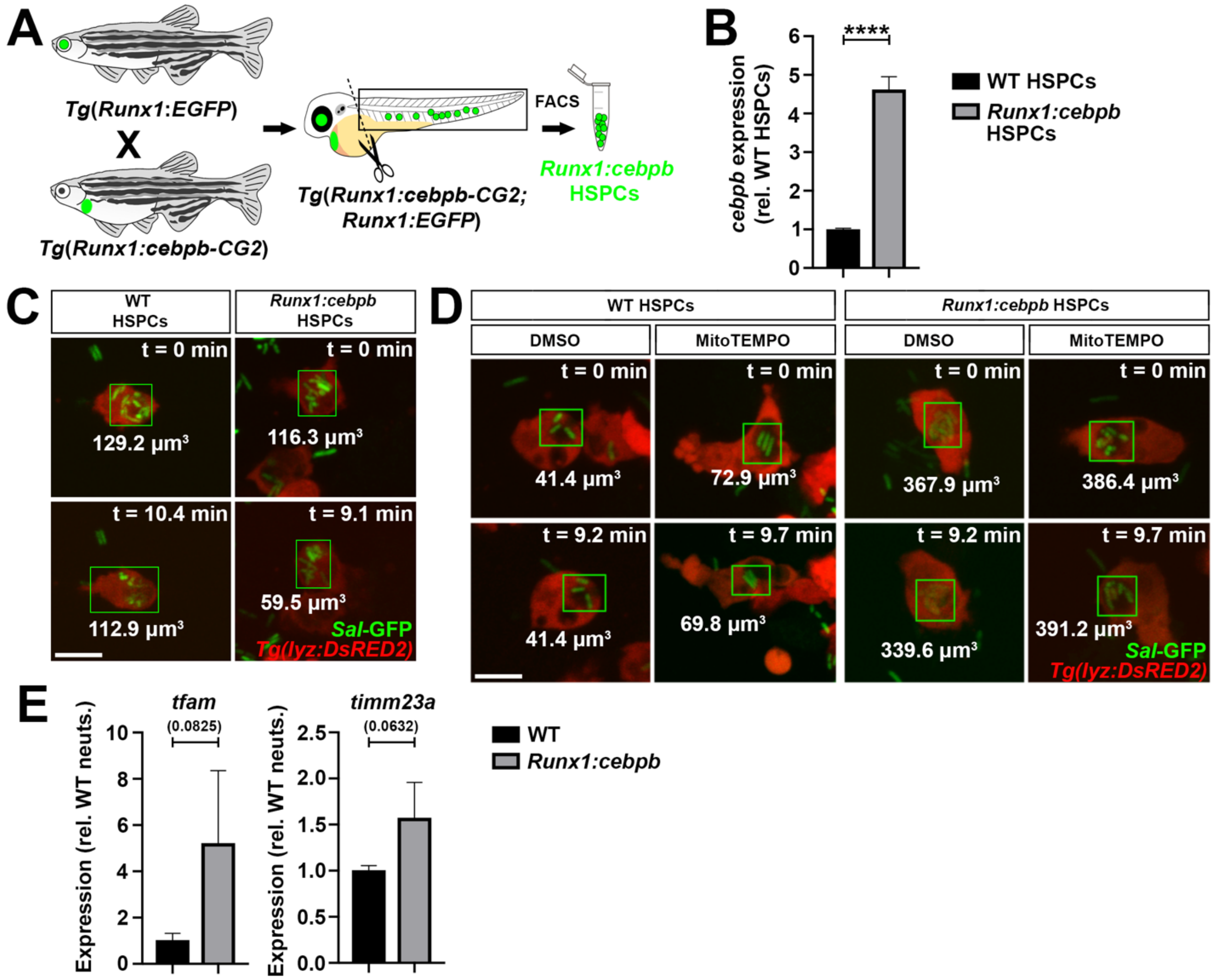

Fig. S7. Confirmation of *cebpb* expression within HSPCs of *Tg(Runx1:cebpb-CG2)* larvae, confocal imaging of neutrophils within control and MitoTEMPO-treated *Tg(Runx1:cebpb-CG2;lyz:DsRED2)* larvae and expression of DEGs of interest *tfam* and *timm23a* within neutrophils isolated from infected *Tg(Runx1:cebpb-CG2;lyzDsRED2)* larvae. (**A**) Schematic illustrating strategy to FACS isolate *Runx1:cebpb*-expressing HSPCs from the dissected trunks of *Tg(Runx1:cebpb-CG2;Runx1:EGFP)* larvae. (**B**) Expression of *cebpb* within WT and *Runx1:cebpb*-expressing HSPCs FACS-isolated from the dissected trunks of 3 dpf *Tg(Runx1:EGFP)*/WT and *Tg(Runx1:cebpb-CG2;Runx1:EGFP)* larvae, respectively, as detected by qPCR (in biological triplicate). (**C**) Frame shots from live time-lapse confocal imaging of neutrophils within *Tg(lyzDsRED2)*/WT and *Tg(Runx1:cebpb-CG2;lyz:DsRED2)* larvae, showing volumes of intracellular *Sal*-GFP at the beginning (t=0) and end of the time-lapse experiment. (**D**) Frame shots from live time-lapse confocal imaging of neutrophils within DMSO- and MitoTEMPO-treated *Tg(lyzDsRED2)*/WT and *Tg(Runx1:cebpb-CG2;lyz:DsRED2)* larvae, showing volumes of intracellular *Sal*-GFP at the beginning (t=0) and end of the time-lapse experiment. (**E**) Expression of *tfam* and *timm23a* within neutrophils FACS-isolated from infected *Tg(lyzDsRED2)*/WT and *Tg(Runx1:cebpb-CG2;lyzDsRED2)* larvae (as shown in Fig. 6G), as detected by qPCR (in biological triplicate). *P* values are shown in brackets. Error bars, mean ± SD; **** *P* <0.0001; unpaired Student's t-test (**B** and **E**). Scale bars, 10 µm.
